# Chlorophyll‐Derived Yellow Phyllobilins of Higher Plants as Medium‐Responsive Chiral Photoswitches

**DOI:** 10.1002/anie.201609481

**Published:** 2016-11-28

**Authors:** Chengjie Li, Klaus Wurst, Steffen Jockusch, Karl Gruber, Maren Podewitz, Klaus R. Liedl, Bernhard Kräutler

**Affiliations:** ^1^ Institute of Organic Chemistry and Centre of Molecular BiosciencesUniversity of Innsbruck6020InnsbruckAustria; ^2^ Institute of General, Inorganic & Theoretical ChemistryUniversity of InnsbruckAustria; ^3^ Department of ChemistryColumbia UniversityNew YorkUSA; ^4^ Institute of Molecular BiosciencesUniversity of GrazAustria; ^5^ Institute of General, Inorganic & Theoretical Chemistry and Centre of Molecular BiosciencesUniversity of InnsbruckAustria

**Keywords:** cycloaddition, hydrogen bonds, photoisomerization, plant pigments, self-assembly

## Abstract

The fall colors are signs of chlorophyll breakdown, the biological process in plants that generates phyllobilins. Most of the abundant natural phyllobilins are colorless, but yellow phyllobilins (phylloxanthobilins) also occur in fall leaves. As shown here, phylloxanthobilins are unique four‐stage photoswitches. Which switching mode is turned on is controlled by the molecular environment. In polar media, phylloxanthobilins are monomeric and undergo photoreversible *Z*/*E* isomerization, similar to that observed for bilirubin. Unlike bilirubin, however, the phylloxanthobilin *Z* isomers photodimerize in apolar solvents by regio‐ and stereospecific thermoreversible [2+2] cycloadditions from self‐assembled hydrogen‐bonded dimers. X‐ray analysis revealed the first stereostructure of a phylloxanthobilin and its hydrogen‐bonded self‐templating architecture, helping to rationalize its exceptional photoswitch features. The chemical behavior of phylloxanthobilins will play a seminal role in identifying biological roles of phyllobilins.

Chlorophyll (Chl) breakdown was a strikingly elusive biological phenomenon until about 25 years ago.^1^ As we know now, this biologically controlled global process generates large amounts of bilin‐type Chl catabolites, so‐called phyllobilins (PBs; see Figure 1).^2^ However, the detection of PBs has been challenging, as, in contrast to all expectations,^3^ de‐greening leaves and ripening fruit mostly accumulate PBs belonging to the colorless phylloleucobilins.^2^, ^4^ Recently, colored, natural Chl‐derived PBs (phyllochromobilins)^2^ were also discovered, which feature similar structures as the ubiquitous colored heme‐derived bilins,^5^ such as bilirubin (**BR**)^6^ and phytochromobilin (**PΦB**; Figure 1).^5^ **PΦB** plays key roles in plants as a light receptor in the photoregulation of gene expression,^7^ which helps plants to adapt to solar light, which is essential for their existence and survival.^8^ In this crucial function, **PΦB** acts as a *Z*/*E* photoswitch.^9^ Hence, the structurally related phyllochromobilins have been considered to be candidates for roles in plants similar to those of (“hemo”)bilins, although a biological function for the globally abundant Chl‐derived PBs is yet unknown.^2^

**Figure 1:**
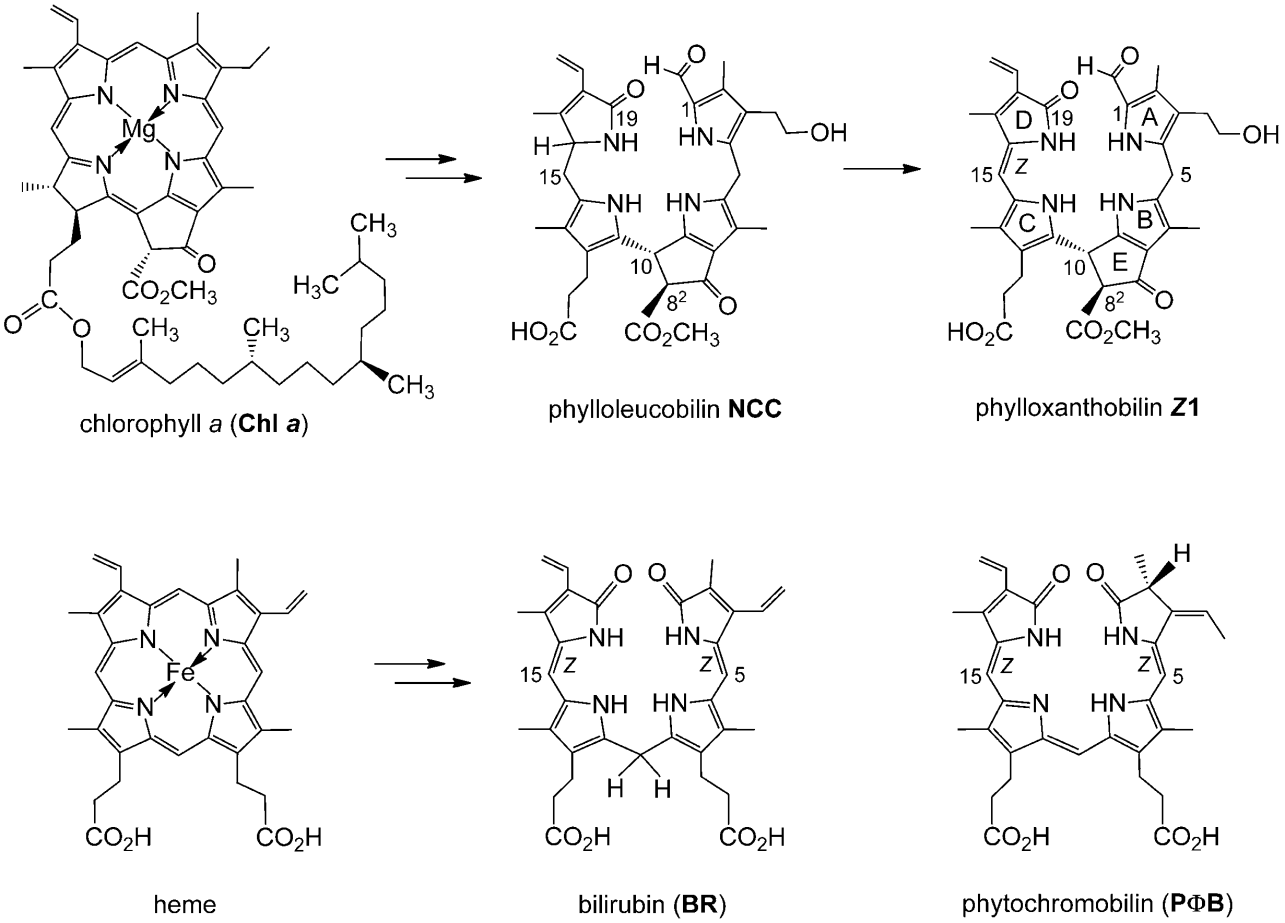
Two important natural degradation pathways to bilins. Top: Breakdown of chlorophyll furnishes phyllobilins (PBs), such as the colorless phylloleucobilin **NCC** and the yellow phylloxanthobilin ***Z*** **1**. Bottom: Heme breakdown leads to structurally related (hemo)bilins, such as bilirubin (**BR**) and phytochromobilin (**PΦB**), shown here as the *Z* isomers.

Herein, we describe the remarkable multiple‐stage photoswitch behavior of yellow Chl catabolites (phylloxanthobilins, PxBs) of higher plants^10^ (Figure 2). The PxB ***Z*** **1** is generated in fall leaves at a late stage of Chl breakdown by oxidation of phylloleucobilin **NCC** (Figure 1).^2^ Compound ***Z*** **1** and its double‐bond isomer ***E*** **1** are both found in leaves,^2^, ^10^, ^11^ where ***Z*** **1** is generated first,^12^ and the occurrence of ***E*** **1** is likely a consequence of *Z*/*E* isomerization upon in vivo exposure of ***Z*** **1** to sunlight (Figure 2). The photo‐induced interconversion of ***Z*** **1** and ***E*** **1** also readily takes place in polar solvents.^11^

**Figure 2:**
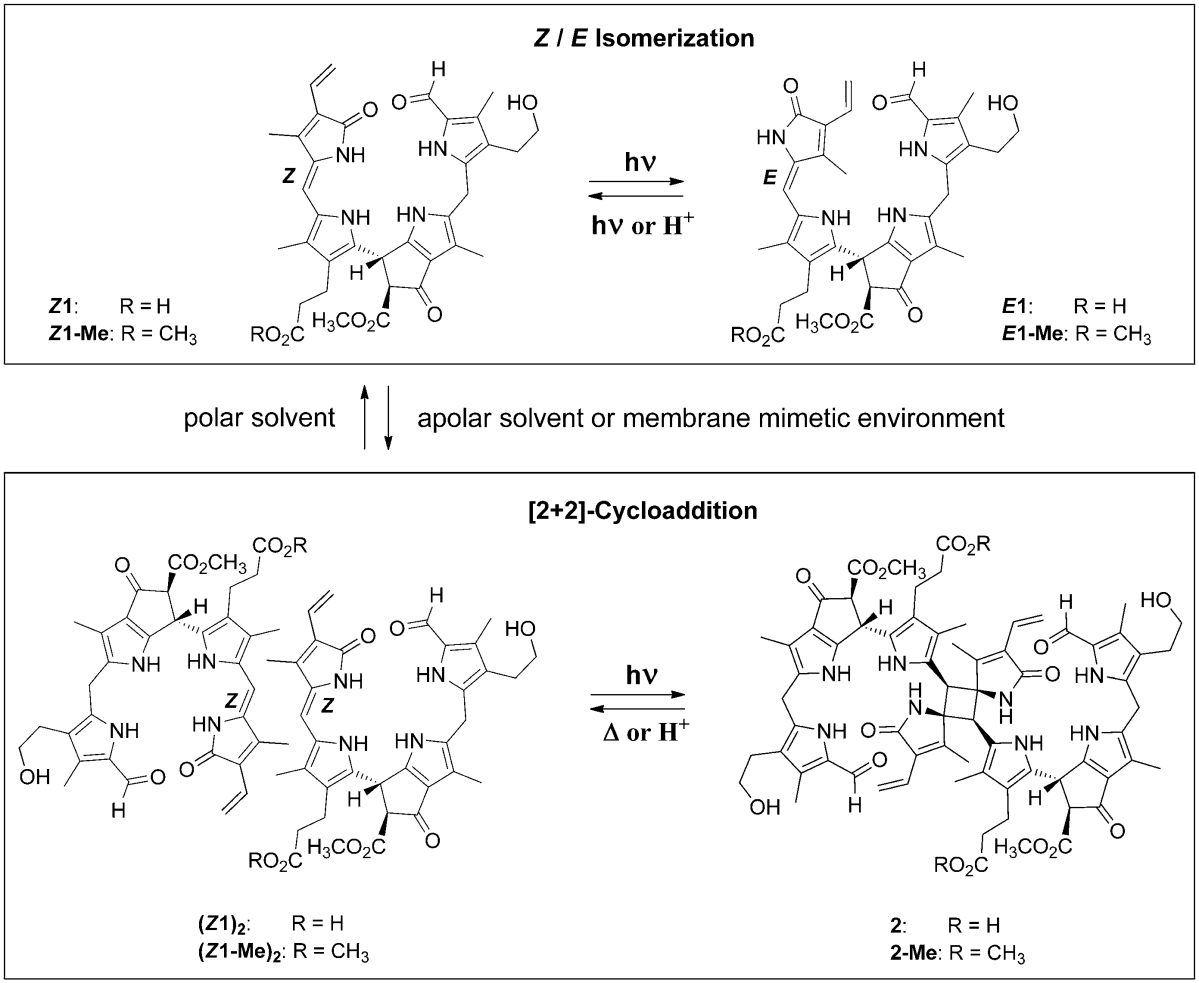
The natural phylloxanthobilin ***Z*** **1** and its methyl ester ***Z*** **1‐Me** behave as medium‐responsive photoswitches. In polar solvents, the *Z* and *E* isomers photointerconvert reversibly, but in apolar solvents or in membrane‐mimetic micellar solutions, ***Z*** **1** and ***Z*** **1‐Me** preorganize by hydrogen‐bonded self‐assembly and undergo [2+2] photocycloadditions to the *C*_2_‐symmetric octapyrroles **2** and **2‐Me**, respectively.

The structures of the yellow tetrapyrroles ***Z*** **1** and ***E*** **1** (and of ***Z*** **1‐Me**) were deduced from spectroscopic data.^10^, ^11^, ^13^ In our study, single crystals of ***Z*** **1‐Me** were obtained that provided the basis for an unprecedented analysis of a chiral phylloxanthobilin by X‐ray crystallography, applying intrinsic phasing and refinement against F^2^ values using the program SHELXTL^14^ for the assignment of the absolute stereostructure (Figure 3; see also the Supporting Information, Tables S1 and S2 and Figures S1 and S2). The crystal analysis of ***Z*** **1‐Me** confirmed the NMR‐derived structure of ***Z*** **1**,^10^ including the lactam function of ring D, and it revealed a high double‐bond character for the adjacent *Z*‐configured C15=C16 bond. Furthermore, it established the *R* and *S* configurations at carbon atoms C10 and C8^2^, respectively, of ***Z*** **1‐Me** (and of ***Z*** **1**).^10^ This absolute configuration at C10 and C8^2^ of ***Z*** **1** was previously proposed on the basis of the tentatively assigned structure of its natural precursor, phylloleucobilin **NCC** (Figure 1).^2^, ^15^

**Figure 3:**
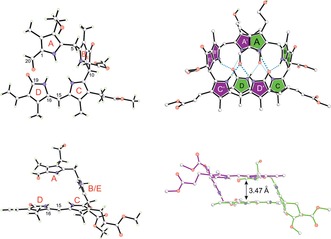
***Z*** **1‐Me** crystallizes as an intertwined homochiral hydrogen‐bonded dimer, shown in ball‐and‐stick representation. This chiral linear tetrapyrrole arranges in a *C*_2_‐symmetric double‐decker structure, in which the π‐stacked main bilirubin‐type chromophores (rings C and D) of both monomers are placed on top of each other (as highlighted on the right side), with their C15=C16 double bonds in close proximity.

In the crystal, ***Z*** **1‐Me** adopts a double‐decker conformation owing to its characteristic B/E ring section, which acts as a spacer (Figure 3). ***Z*** **1‐Me** further associates into an intertwined, hydrogen‐bonded, and nearly *C*_2_‐symmetric homodimer. In these non‐covalent chiral dimers, two molecules of ***Z*** **1‐Me** are connected by eight hydrogen bonds and by additional π‐stacking contacts between the almost parallel planes of their bilirubin‐type main chromophores. The short C15=C16 bonds of two ***Z*** **1‐Me** molecules are placed nearly on top of each other, leading to intermodular C15–C16′/C15′–C16 distances of 3.63 and 3.75 Å, respectively (Figure S2). Aside from the dominant homodimer **(*Z*1‐Me)_2_**, a heterodimer ***Z*** **1‐Me**/*epi*‐***Z*** **1‐Me** (15 %) is also present in the crystal, in which the C8^2^ epimer *epi*‐***Z*** **1‐Me** (see pink star in Figure 4) is paired up with ***Z*** **1‐Me**.

**Figure 4:**
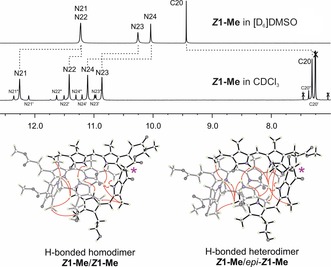
The structure of ***Z*** **1‐Me** is strongly solvent‐dependent. 500 MHz ^1^H NMR spectra at 25 °C indicate that ***Z*** **1‐Me** is present in monomeric form in [D_6_]DMSO (top) and as extensively hydrogen‐bonded and π‐stacked dimers **(*Z*1‐Me)**_2_ in CDCl_3_ (middle), with a structure similar to the one observed in the crystal. The diagnostic signals of protons at low field, bound to N atoms or to the formyl C20 atom, are shown. Resonance assignments and intermodular NOE correlations observed in CDCl_3_ solutions of ***Z*** **1‐Me** are visualized based on the crystal structure. The resonances originate from the major hydrogen‐bridged homodimer **(*Z*1‐Me)**_2_ and the minor epimeric heterodimer (***Z*** **1‐Me**/*epi‐**Z*** **1‐Me**), which contributes the eight satellite‐like bands. The epimerization site C8^2^ is indicated with a pink star.

In polar solvents ([D_6_]DMSO), ***Z*** **1** and ***Z*** **1‐Me** exhibited NMR spectral features of monomeric PBs.^10^, ^13^ In contrast, ^1^H NMR data of solutions of ***Z*** **1** in CDCl_3_/dioxane (9:1) and of ***Z*** **1‐Me** in CDCl_3_ were not compatible with a monomeric structure (Figure 4; see also Figures S3 a, b, S4 a–c and Tables S3–4). Instead, nuclear Overhauser effect (NOE) correlations and the deduced spatial arrangements indicated the association of ***Z*** **1‐Me** into hydrogen‐bonded dimers **(*Z*1‐Me)_2_**. The solution structure of ***Z*** **1‐Me** in CDCl_3_ was clarified with the help of the crystal structure to rationalize its unexpected ^1^H NMR spectrum (Figure 4). In the spectrum of ***Z*** **1‐Me**, the four main resonances at low field were individually assigned to the four N‐bonded H atoms of the predominant symmetric homodimer **(*Z*1‐Me)_2_**. These resonances were superimposed by a set of eight additional signals (with 15 % intensity) that were due to the minor unsymmetric heterodimer ***Z*** **1‐Me**/*epi*‐***Z*** **1‐Me**.

Hence, depending on the solvent polarity, ***Z*** **1‐Me** and ***Z*** **1** predominantly exist in solution as monomers or as hydrogen‐bonded dimers, which was consistently corroborated by UV/Vis, fluorescence, and CD spectroscopy (Figure 5 and Figure S5 a–d), and by the dual path photochemistry of the two PxBs (see below). The UV/Vis spectra of solutions of ***Z*** **1**, ***E*** **1**, and ***Z*** **1‐Me** in polar solvents^10^, ^11^, ^13^ and in CHCl_3_ were similar, with slight shifts of the absorption maxima and the appearance of shoulders at lower transition energies in the latter (Figure 5 and Figure S5 b). However, the CD spectra of ***Z*** **1** and ***Z*** **1‐Me** showed a pronounced solvent dependence (Figure S5 d). Solutions of ***Z*** **1‐Me** in MeOH exhibited weak CD effects near 340 nm,^13^ but a strong positive Cotton effect was observed near *λ*=350 nm in CHCl_3_,^16^ diagnostic of a P‐type arrangement^6^ of the pair of A rings in the hydrogen‐bonded dimer **(*Z*1‐Me)_2_**, as found in the crystal.

**Figure 5:**
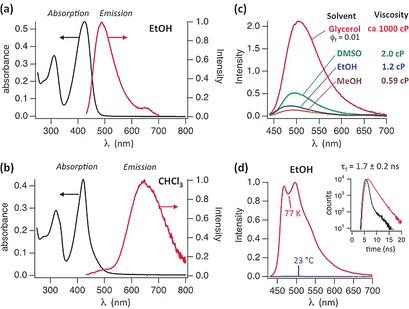
Solvent‐dependent UV/Vis and fluorescence spectra of ***Z*** **1‐Me**. a) UV/Vis and fluorescence spectra of ***Z*** **1‐Me** (10 μm) in EtOH exhibit the spectral features of monomers. b) UV/Vis and fluorescence spectra of ***Z*** **1‐Me** in CHCl_3_ (0.4 mm) support the presence of hydrogen‐bonded dimers and excimer luminescence. c) Fluorescence spectra of ***Z*** **1‐Me** in polar solvents with different viscosities at room temperature (adjusted to matching absorbances at *λ*_ex_=424 nm); in glycerol a fluorescence quantum yield of *Φ*_f_=0.01 was determined. d) Fluorescence spectra of ***Z*** **1‐Me** in EtOH at 77 K and at room temperature. Inset: Fluorescence lifetime of ***Z*** **1‐Me** of about 1.7 ns (at *λ*=480 nm, red trace; the instrument response function is shown in black). In toluene (at 77 K), the fluorescence spectrum featured a band at *λ*_em_=660 nm with a lifetime of about 10 ns, which is consistent with the excimer emission of **(*Z*1‐Me)_2_** (Figure S5 a).

Fluorescence spectra of ***Z*** **1** and ***Z*** **1‐Me** displayed a strong solvent dependence, similar to that observed for their CD spectra. In polar solvents at 23 °C, the fluorescence of ***Z*** **1‐Me** was weak, and its intensity was strongly viscosity‐dependent (see Figure 5), which is characteristic of a deactivation of the excited singlet state by a rapid twist and eventual *Z*/*E* isomerization around a critical C=C bond.^5^, ^17^ The fluorescence emission maxima in EtOH and CHCl_3_ were observed near *λ*=480 and 650 nm, respectively. The former situation suggests emission from the monomeric state of ***Z*** **1‐Me**, the latter excimer emission from the preformed non‐covalent dimer **(*Z*1‐Me)_2_**. In CHCl_3_, the ratio between excimer and monomer emission of ***Z*** **1‐Me** was concentration‐dependent and increased from about 3 at 10 μm to 17 at 0.4 mm. Absorption, CD, and fluorescence spectra indicated strongly solvent‐dependent equilibria between ***Z*** **1‐Me** and **(*Z*1‐Me)_2_**. An association constant of *K*_a_≈5×10^4^ m ^−1^ was calculated from the concentration‐dependent absorption bands of ***Z*** **1‐Me**/**(*Z*1‐Me)_2_** in acetonitrile (Figure S5 c). In CHCl_3_ (or toluene), the absorption bands were effectively concentration‐insensitive, indicating the predominant presence of the hydrogen‐bonded dimer **(*Z*1‐Me)_2_** (i.e., *K*_a_>10^5^ m ^−1^). Conversely, the concentration insensitivity of the spectra of ***Z*** **1** and ***Z*** **1‐Me** in MeOH suggested a much lower value of *K*_a_<10^3^ m ^−1^ in this solvent.

Irradiation of a solution of ***Z*** **1** and ***Z*** **1‐Me** in MeOH with a fluorescence lamp caused efficient (photoreversible) conversion into their less stable isomers ***E*** **1** and ***E*** **1‐Me**, respectively, reaching a steady state near 50 % conversion (Figure S6 a). Irradiation at *λ*=394 or 435 nm of ***Z*** **1** in acetonitrile solution gave ***E*** **1** in an estimated quantum yield of 0.2 (from ferrioxalate actinometry). The reverse *E* to *Z* isomerization also occurred readily upon illumination (Figure S6 a) or in protic solvents (in the dark). Storage of a solution of ***E*** **1‐Me** in CHCl_3_/1 % acetic acid at room temperature in the dark for 5 h thus led to 95 % conversion into ***Z*** **1‐Me** (Figure S6 b). The *E* isomer ***E*** **1‐Me** was available through preparative photoisomerization of ***Z*** **1‐Me**, and its structure was deduced by detailed spectroscopic analysis (Figure S6 b–e and Table S3).

In marked contrast to the situation in MeOH, exposure of a solution of ***Z*** **1‐Me** in CDCl_3_ to a fluorescence lamp at 0 °C caused the disappearance of the 420 nm absorption (Figure 6), and resulted in clean regio‐ and stereoselective conversion into the colorless octapyrrolic photodimer **2‐Me** (>95 %, <2 % of ***E*** **1‐Me** formed; see Figure S8 a). However, no evidence for a similar dimer formation was found for ***E*** **1‐Me**. Indeed, when a solution of ***E*** **1‐Me** in CDCl_3_ was exposed to light, ***E*** **1‐Me** first isomerized to ***Z*** **1‐Me**, which subsequently underwent photodimerization to **2‐Me** (Figure S7). The covalent *C*_2_‐symmetric [2+2] photodimer **2‐Me** was rather stable at 2 °C, and its structure could be thoroughly investigated by NMR spectroscopy. The two halves of the *C*_2_‐symmetric octapyrrole **2‐Me** are connected via a cyclobutane core, which is characterized by methine resonances at *δ*=4.33 ppm (^1^H) and 43.7 ppm (^13^C), and by a ^13^C resonance of a quaternary carbon atom at *δ*=73.1 ppm. The hydrogen bonds seen in the crystal and in CDCl_3_ solution of the homodimer **(*Z*1‐Me)_2_** appear to be retained in **2‐Me** (Figure S8 a–d and Tables S3 and S5).

**Figure 6:**
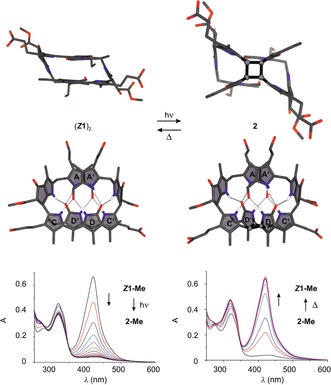
Phylloxanthobilin ***Z*** **1** dimerizes in CHCl_3_ in a thermoreversible [2+2] photocycloaddition to octapyrrole **2**. Comparison of the calculated structures of the preorganized, self‐templating, hydrogen‐bonded, and π‐stacked homodimer **(*Z*1)_2_** (top left) and **2** (top right) indicates complete retention of the hydrogen‐bonding network. Photolysis of yellow ***Z*** **1‐Me** in CHCl_3_ at 0 °C generates **2‐Me** (bottom left). Photodimerization is reversed by thermolysis of **2‐Me** at room temperature or 50 °C (bottom right).

The UV/Vis spectrum of the dimer **2‐Me** exhibited an absorption maximum near *λ*=320 nm (due to its formylpyrrole unit), which is consistent with the interruption of the main chromophore of ***Z*** **1‐Me**. The strong Cotton effect near *λ*=330 nm in the CD spectrum of **2‐Me** (Figure S8 e) suggests a P‐type arrangement of the A/A′ rings in **2‐Me** similar to that found in the structure of the hydrogen‐bonded dimer **(*Z*1‐Me)_2_** (Figure 3 and Figure 6). The structures of the monomeric PxBs ***E*** **1‐Me** and ***Z*** **1‐Me** as well as of the hydrogen‐bonded dimer **(*Z*1‐Me)_2_** and the covalent photodimer **2‐Me** were calculated with density functional theory (BP86/def2‐TZVP/D3; Figure 6 and Figure S9 a, b). The computed structural models of the PxBs **(*Z*1‐Me)_2_** and **2‐Me** featured intramolecular (inter‐ and intramodular) distances that were consistent with the observed NOEs and with the proposed network of eight hydrogen bonds that appears to be conserved during the interconversion of **(*Z*1‐Me)_2_** and **2‐Me**.

The spectral and photochemical properties of ***Z*** **1** are also medium‐dependent in aqueous solutions. At pH 7, ***Z*** **1** is poorly soluble and largely monomeric. ***Z*** **1** undergoes clean photoinduced *Z*/*E* isomerization, reaching an apparent photostationary state at ***E*** **1**/***Z*** **1**≈1:1, and ***E*** **1** reverts back to ***Z*** **1** in the dark (71 h, 23 °C; Figure S10 a, b). However, in aqueous micellar solutions of sodium dodecylsulfonate (SDS), ***Z*** **1** dissolved to a higher concentration, and epimerized partly to its C8^2^ stereoisomer *epi*‐***Z*** **1**. The SDS solution had a mean occupancy of about two PxB molecules per micelle, based on an aggregation number of about 70.^18^ ^1^H NMR spectra and other data revealed the predominance of hydrogen‐bonded, π‐stacked dimers, **(*Z*1)_2_** and ***Z*** **1**/*epi*‐***Z*** **1** (Figure S3 b). Upon irradiation at 23 °C, the micellar PxB mixture underwent effective photodimerization to homodimer **2** and heterodimer *epi*‐**2** (in a ratio of about 1.2:1). A photostationary state was reached at about 70 % conversion owing to effective thermal cycloreversion of **2** or *epi*‐**2** into ***Z*** **1** or *epi*‐***Z*** **1** (Figure S11 a–c). Likewise, when natural ***Z*** **1** was irradiated in CHCl_3_/dioxane (99:1) at 0 °C for 2 h, dimer **2** was generated in >90 % yield, as determined by ^1^H NMR and HPLC analysis (Figure S12 a, b).

Thermolytic [2+2] cycloreversion of the thermally labile covalent dimer **2‐Me** at room temperature cleanly furnished the hydrogen‐bonded dimer **(*Z*1‐Me)_2_** (Figure 6). In acid‐free CHCl_3_, the colorless, covalent photodimer **2‐Me** displayed a half‐life of 2.6 h at 50 °C and about 50 h at 30 °C (Figure S13). Opening of the cyclobutane ring of **2‐Me** in CHCl_3_ is strongly accelerated upon adding 0.2 % of trifluoroacetic acid. Likewise, in aqueous micellar SDS solution at pH 7, ring opening of the covalent photodimer **2** is about twenty times faster than in CHCl_3_ (Figures S8 a and S11 c). A stepwise polar mechanism presumably accounts for this cleavage reaction, similar to those that promote thermal cyclobutane ring opening in related heterocyclic natural products.^19^, ^20^ Complete cycloreversion of **2‐Me** into two molecules of yellow ***Z*** **1‐Me** is consistent with exergonic cleavage of the four‐membered ring, which was supported by quantum‐chemical calculations. Interestingly, the same cleavage products would be obtained from the ring‐opening reaction independent of whether it involves the two bonds made by the photodimerization or the ring positions that originate, in a formal sense, from the original C15=C16 bond. The retained hydrogen‐bond network and the different bond lengths calculated for the C−C bonds of the cyclobutane core (1.57 vs. 1.62 Å) support the hypothesis that cleavage of the same two bonds that were formed in the cycloaddition reaction is preferred.

Phylloxanthobilins (PxBs) are ubiquitous yellow phyllobilins^2^ that are detected in senescent leaves as minor^10^, ^11^, ^21^ or as major products^22^ of Chl degradation. The PxBs ***Z*** **1** and ***E*** **1** are medium‐responsive chiral photoswitches^23^ that undergo either rapid and specific *Z*/*E* isomerization or efficient regio‐ and stereospecific photoinduced [2+2] cyclodimerization. This latter process is due to the assembly of hydrogen‐bonded dimers, as observed in the crystals of ***Z*** **1‐Me**, featuring the unique “hand‐shake” motif (Figure 3). Supramolecular preorganization of ***Z*** **1** and photoinduced [2+2] cyclodimerization to **2** also occur in micellar solutions, which provide hydrophobic “cages” and a molecular confinement17b, 24 mimicking medium effects present in the compartmentalized and membraneous structures of cells.^25^ The chameleon‐like medium‐responsive multistage photochromic activity of phylloxanthobilins ***Z*** **1** and ***E*** **1** is therefore likely to be relevant in leaves, their natural environment.

PxBs feature the same main chromophore as bilirubin (**BR**), the natural yellow “animal pigment” from heme breakdown.^5^, ^6^ However, the aggregation behavior of ***Z*** **1** differs strongly from that of **BR**, which crystallizes as a hydrogen‐bonded monomer in a “roof tile” structure.^6^, ^26^ The native *Z*,*Z* form of (monomeric) **BR** undergoes photoisomerization to its more water‐soluble *E*,*Z* isomer, a process used for the common medical treament of neonatal jaundice.17a **BR** also undergoes other photochemical reactions with low selectivity but its [2+2] photocyclization is unknown.^6^, 9b Indeed, this type of photoreaction appears to be a specific consequence of the self‐templated double‐decker structure of ***Z*** **1** and ***Z*** **1‐Me**, spanned by the characteristic B/E ring moiety, and is unprecedented for heme‐derived bilins, which lack this structural motif.^6^ A range of unsaturated compounds undergo [2+2] photocyclodimerization,17b a likely photoprocess at properly preorganized C=C double bonds.^24^

In living organisms, (heme‐derived) bilins play important roles.^5^, ^6^, ^7^, ^8^, ^9^ When excited by visible light, protein‐bound phytochromobilin (**PΦΒ**) undergoes *Z*/*E* isomerization of its substituted C15=C16 bond,^5^, 9b, 17b which constitutes the specific basis for the function of this natural photoswitch as a molecular photoregulator in phytochromes of higher plants.9a Cyanobacteriochromes are related natural bilin‐based photoswitches in cyanobacteria.^27^ The specific *Z*/*E* photoswitch behavior of the Chl‐derived phylloxanthobilins **Z1** and ***E*** **1** may represent a biologically relevant functional mimicry of the important photoregulatory roles of related bilins. Indeed, photoswitches are central elements of most natural photoregulatory components in life processes^8^, ^28^, ^29^ as well as in photopharmacological,^30^ optogenetic,^31^ and technical devices.^32^ In this context, the here described natural Chl‐derived PxBs may be considered as remarkable medium‐responsive, chiral four‐stage photoswitches28a that are present in plant cells. Hence, we are particularly intrigued by the question as to how PxBs and other phyllochromobilins interact with the photoregulatory system of plants.

Phylloxanthobilins (PxBs) and their pink relatives (phylloroseobilins)^33^ are natural pigments that absorb solar light, act as sun screens, and contribute to pigmentation in senescent leaves.^2^, ^11^, ^21^ Such phyllochromobilins are rather hydrophobic and are expected to be largely bound to proteins or membranes, possibly playing a role in inhibiting autoxidation. In leaf cells, phylloxanthobilin ***Z*** **1** also undergoes light‐induced isomerization to ***E*** **1**. Alternatively, ***Z*** **1** may photodimerize in the apolar environment of biomembranes to the unstable photodimer **2**, which, however, has not yet been detected in leaves.

The fascinating chemistry of the phyllochromobilins ***Z*** **1** and ***E*** **1** points towards beneficial functions in plants. Phyllobilins are conspicuously abundant linear tetrapyrroles with an extraordinary structure‐based potential for biological roles. Hence, the elucidation of a biological function of Chl‐derived PBs is indeed truly challenging.^2^ Their possible relevance in the context of photoregulation in plants is particularly intriguing.

## Experimental Section

See the Supporting Information for details on the materials, instruments, synthetic procedures, spectroscopy, photochemical and computational studies, as well as X‐ray crystallography. Crystals of ***Z*** **1‐Me** grew in the orthorhombic space group *P*2_1_2_1_2_1_. CCDC 1500880 contains the supplementary crystallographic data for this paper. These data are provided free of charge by The Cambridge Crystallographic Data Centre.

*Dedicated to Professor Christoph Kratky on the occasion of his 70th birthday*

## Supporting information

As a service to our authors and readers, this journal provides supporting information supplied by the authors. Such materials are peer reviewed and may be re‐organized for online delivery, but are not copy‐edited or typeset. Technical support issues arising from supporting information (other than missing files) should be addressed to the authors.

Supplementary

## References

[1a] G. A. F. Hendry , J. D. Houghton , S. B. Brown , New Phytol. 1987, 107, 255–302; 10.1111/j.1469-8137.1987.tb00181.x 33873847

[1b] S. Hörtensteiner , B. Kräutler , Biochim. Biophys. Acta Bioenergetics 2011, 1807, 977–988. 10.1016/j.bbabio.2010.12.007 21167811

[2a] B. Kräutler , Chem. Soc. Rev. 2014, 43, 6227–6238; 24898066 10.1039/c4cs00079j

[2b] B. Kräutler , Angew. Chem. Int. Ed. 2016, 55, 4882–4907; 10.1002/anie.201508928 PMC4950323 26919572

[3] P. Matile , Chimia 1987, 41, 376–381.

[4a] B. Kräutler , B. Jaun , K. Bortlik , M. Schellenberg , P. Matile , Angew. Chem. Int. Ed. Engl. 1991, 30, 1315–1318;

[4b] T. Müller , M. Ulrich , K.-H. Ongania , B. Kräutler , Angew. Chem. Int. Ed. 2007, 46, 8699–8702; 10.1002/anie.200703587 PMC2912502 17943948

[5] H. Falk , Chemistry of Linear Oligopyrroles and Bile Pigments, Springer, Vienna, 1989.

[6] D. A. Lightner , Bilirubin: Jekyll and Hyde Pigment of Life, Springer, Vienna, 2013. 10.1007/978-3-7091-1637-1_1 24597429

[7] N. C. Rockwell , J. C. Lagarias , Plant Cell 2006, 18, 4–14. 16387836 10.1105/tpc.105.038513 PMC1323480

[8a] Photoprotection, Photoinhibition, Gene Regulation and Environment (Eds.: B. Demmig-Adams, W. Adams, A. Mattoo), Springer, Dordrecht, 2006;

[8b] A. Möglich , X. J. Yang , R. A. Ayers , K. Moffat , Annu. Rev. Plant Biol. 2010, 61, 21–47. 20192744 10.1146/annurev-arplant-042809-112259

[9a] E. S. Burgie , R. D. Vierstra , Plant Cell 2014, 26, 4568–4583; 25480369 10.1105/tpc.114.131623 PMC4311201

[9b] S. E. Braslavsky , A. R. Holzwarth , K. Schaffner , Angew. Chem. Int. Ed. Engl. 1983, 22, 656–674;

[10] S. Moser , M. Ulrich , T. Müller , B. Kräutler , Photochem. Photobiol. Sci. 2008, 7, 1577–1581. 19037512 10.1039/b813558d PMC2906697

[11] M. Ulrich , S. Moser , T. Müller , B. Kräutler , Chem. Eur. J. 2011, 17, 2330–2334. 21308817 10.1002/chem.201003313 PMC3072522

[12] C. Vergeiner , M. Ulrich , C. Li , X. Liu , T. Müller , B. Kräutler , Chem. Eur. J. 2015, 21, 136–149. 25382809 10.1002/chem.201404783 PMC4517098

[13] C. Li , B. Kräutler , J. Porphyrins Phthalocyanines 2016, 20, 388–396.

[14] Bruker AXS Inc. Madison, Wisconsin, USA, **2014**.

[15a] M. Oberhuber , J. Berghold , K. Breuker , S. Hörtensteiner , B. Kräutler , Proc. Natl. Acad. Sci. USA 2003, 100, 6910–6915; 12777622 10.1073/pnas.1232207100 PMC165803

[15b] M. Oberhuber , J. Berghold , B. Kräutler , Angew. Chem. Int. Ed. 2008, 47, 3057–3061; 10.1002/anie.200705330 PMC2912497 18327758

[16] Circular dichroism—Principles and applications (Eds.: K. Nakanishi, N. Berova, R. W. Woody), Wiley-VCH, New York, 1994.

[17a] D. A. Lightner , A. F. McDonagh , Acc. Chem. Res. 1984, 17, 417–424;

[17b] N. J. Turro , V. Ramamurthy , J. C. Scaiano , Modern Molecular Photochemistry of Organic Molecules, University Science Books, Sausalito, 2010.

[18] J. H. Fendler , E. J. Fendler , Catalysis in Micellar and Macromolecular Systems, Academic Press, New York, 1975.

[19] A. Skiredj , M. A. Beniddir , D. Joseph , G. Bernadat , L. Evanno , E. Poupon , Synthesis 2015, 47, 2367–2376.

[20] P. S. Baran , D. P. O'Malley , A. L. Zografos , Angew. Chem. Int. Ed. 2004, 43, 2674–2677; 10.1002/anie.200453937 18629987

[21] M. Scherl , T. Müller , B. Kräutler , Chem. Biodiversity 2012, 9, 2605–2617. 10.1002/cbdv.201200203 PMC3586658 23161638

[22] D. Wakana , H. Kato , T. Momose , N. Sasaki , Y. Ozeki , Y. Goda , Tetrahedron Lett. 2014, 55, 2982–2985.

[23] B. L. Feringa , R. A. van Delden , N. Koumura , E. M. Geertsema , Chem. Rev. 2000, 100, 1789–1816. 11777421 10.1021/cr9900228

[24] V. Ramamurthy , J. Sivaguru , Chem. Rev. 2016, 116, 9914–9993. 27254154 10.1021/acs.chemrev.6b00040

[25a] M. Tevini , D. Steinmüller , Planta 1985, 163, 91–96; 24249273 10.1007/BF00395902

[25b] R. R. Wise in The Structure and Function of Plastids (Eds.: R. R. Wise, J. K. Hoober), Springer, Dordrecht, 2006, pp. 3–26.

[26] R. Bonnett , J. E. Davies , M. B. Hursthouse , Nature 1976, 262, 326–328. 10.1038/262326a0 958385

[27] N. C. Rockwell , S. S. Martin , J. C. Lagarias , Photochem. Photobiol. Sci. 2015, 14, 929–941. 25738434 10.1039/c4pp00486h

[28a] B. Feringa , Molecular Switches, Wiley-VCH, Weinheim, 2001;

[28b] K. Gruber , B. Kräutler , Angew. Chem. Int. Ed. 2016, 55, 5638–5640; 10.1002/anie.201601120 27010518

[28c] M. Jost , J. Fernandez-Zapata , M. C. Polanco , J. M. Ortiz-Guerrero , P. Y.-T. Chen , G. Kang , S. Padmanabhan , M. Elias-Arnanz , C. L. Drennan , Nature 2015, 526, 536–541. 26416754 10.1038/nature14950 PMC4634937

[29a] J. M. Ortiz-Guerrero , M. C. Polanco , F. J. Murillo , S. Padmanabhan , M. Elias-Arnanz , Proc. Natl. Acad. Sci. USA 2011, 108, 7565–7570; 21502508 10.1073/pnas.1018972108 PMC3088613

[29b] F. Pennacchietti , A. Losi , X. L. Xu , K. H. Zhao , W. Gärtner , C. Viappiani , F. Cella , A. Diaspro , S. Abbruzzetti , Photochem. Photobiol. Sci. 2015, 14, 229–237. 25358617 10.1039/c4pp00337c

[30] J. Broichhagen , J. A. Frank , D. Trauner , Acc. Chem. Res. 2015, 48, 1947–1960. 26103428 10.1021/acs.accounts.5b00129

[31] T. Fehrentz , M. Schönberger , D. Trauner , Angew. Chem. Int. Ed. 2011, 50, 12156–12182; 10.1002/anie.201103236 22109984

[32a] B. L. Feringa , J. Org. Chem. 2007, 72, 6635–6652; 17629332 10.1021/jo070394d

[32b] W. Gärtner , ChemBioChem 2010, 11, 1649–1652. 20665617 10.1002/cbic.201000302

[33] C. Li , M. Ulrich , X. Liu , K. Wurst , T. Müller , B. Kräutler , Chem. Sci. 2014, 5, 3388–3395.

