# Supplementary material for: Chlorophyll‐Derived Yellow Phyllobilins of Higher Plants as Medium‐Responsive Chiral Photoswitches
Source: Angew Chem Int Ed Engl. 2016 Nov 28;55(51):15760–5. doi: 10.1002/anie.201609481 (PMC5248603; doi:10.1002/anie.201609481)
Supplement: Supplementary file 1 — Supplementary [file ANIE-55-15760-s001.pdf]

Angewandte Chemie
Eine Zeitschrift der Gesellschaft Deutscher Chemiker

Supporting Information

# Chlorophyll-Derived Yellow Phyllobilins of Higher Plants as Medium-Responsive Chiral Photoswitches

*Chengjie Li, Klaus Wurst, Steffen Jockusch, Karl Gruber, Maren Podewitz, Klaus R. Liedl, and Bernhard Kräutler**

anie_201609481_sm_miscellaneous_information.pdf

**Experimental Part.**

*General.* $CHCl_3$, *n*-hexane, dioxane, reagent-grade commercials; MeOH (HPLC grade), and $CH_3CN$ (HPLC grade), from VMR (Leuven, Belgium); water, from Millipore S. A. S. Milli-Q Academic system (18.2 MΩ·cm, Molsheim, France); ACS reagent $KH_2PO_4$ and $K_2HPO_4$, acetic acid (AcOH), trifluoroacidic acid (TFA) and aluminum oxide (activated, basic, Brockmann I) from Sigma-Aldrich (Steinheim, Germany). Sodium dodecyl sulfate (SDS), blotting grade, from Carl Roth (Karlsruhe, Germany). $NH_4OAc$ (≥ 98 %), from Fluka (Buchs, Switzerland). Phylloxanthobilins ***Z*1** and ***Z*1-Me** (see main text) [1-2]. Sep-Pak-C18 Cartridges (Silica-based bonded phase with strong hydrophobicity), from Waters Associates (Milford, USA); reversed phase silica gel (Sepra C18-E, 50µm, 65A), from Phenomenex (Aschaffenburg, Germany). pH-Values, measured with a WTW Sentix 21 electrode, WTW pH535 digital pH meter. Light source for photochemistry: fluorescent lamp (21 W), from Sylvania FHE 21W/T5/830 (London, England).

*Spectroscopy.* UV/Vis: Varian Cary 60 spectrophotometer; $\lambda_{max}$ in nm (log $\varepsilon$). CD-spectra: JASCO J-715 spectropolarimeter; $\lambda_{max}$ and $\lambda_{min}$ in nm ($\Delta\varepsilon$). NMR: Varian UNITY plus 500 and Bruker UltraShield 600 MHz; $\delta$ in ppm with $\delta$ ($CHCl_3$) = 7.26 ppm, $\delta$ ($CD_3SOD_2H$) = 2.50 ppm, coupling constant $J_{HH}$ in Hz; ESI-MS: Finnigan LCQ Classic, ESI source, positive ion-mode, flow rate 2 mL $min^{-1}$, solvent water/MeOH.

*Analytical HPLC*: *Gynkotek* 'high precision pump' *480G* with vacuum on-line degasser, *Gynkotek* diode array detector *DA340*, all chromatograms were taken at room temperature, *Phenomenex*, *ODS-Hypersil* 5µ, 250 × 4.6 mm i.d. pre-column was used with a flow rate 0.5 ml $min^{-1}$; solvent A: 50 mM aq. potassium phosphate (pH 7.0), solvent B: MeOH, solvent C: $H_2O$, solvent D: $NH_4OAc$ (10 mM, pH 6.4); solvent system I (Sosy I): (A/B/C) 0-5 min: 80/20/0; 5-55 min: from 80/20/0 to 30/70/0; 55-60 min: from 30/70/0 to 0/100/0; 60-70 min:

0/100/0; 70-75 min: from 0/100/0 to 80/20/0. Sosy II: (A/B/C) 0-5 min: 60/40/0; 5-15 min: from 60/40/0 to 30/70/0; 15-25 min: from 30/70/0 to 0/100/0; 25-35 min: from 0/100/0; 35-37 min: 0/100/0 to 0/90/10; 37-42 min from 0/90/10 to 60/40/0. Sosy III: (D/B/C) 0-5 min: 60/40/0; 5-15 min: from 60/40/0 to 30/70/0; 15-25 min: from 30/70/0 to 0/100/0; 25-35 min: 0/100/0; 35-37 min: from 0/100/0 to 0/90/10; 37-42 min from 0/90/10 to 60/40/0.

***Photochemical studies.***

Steady-state luminescence spectra were recorded on a Fluorolog-3 fluorometer (HORIBA Jobin Yvon). Fluorescence lifetimes were measured by time correlated single photon counting on an OB920 spectrometer (Edinburgh Analytical Instruments); fluorescence quantum yield was determined in reference to 9,10-diphenylanthracene.

**Computational Details.**

Density functional theory calculations were performed on the monomers, ***Z*1-Me**, ***E*1-Me**, as well as on the dimers, **(*Z*1)$_2$**, **(*Z*1-Me)**$_2$, **2**, and **2-Me**, with the quantum chemical program suite Turbomole.[3] Gas phase structures were fully optimized with the BP86 density functional [4] in combination with the resolution-of-identity [5] and the def2-TZVP basis set [6] plus empirical dispersion corrections of the D3 type by Grimme.[7] Incorporation of empirical dispersion corrections was necessary to obtain the correct distance for the π-stacking of rings C and D, when compared to the experimental (***Z*1-Me**)$_2$ structure. D3 corrections were used for all structure optimizations. The starting structure of **2-Me** was created by manual modification of (***Z*1-Me**)$_2$. As no crystal data was available for **2-Me**, various conformations were generated by Maestro’s conformational search [8] und subsequently optimized with BP86/RI/def2-TZVP/D3. The same procedure was applied to ***Z*1-Me** and ***E*1-Me**.

Solvent effects are neglected because only implicit solvent corrections are feasible and they are expected to be small for non-polar solvents. Reaction energies are electronic energies excluding zero-point and thermal corrections. In agreement with experimental findings ***Z*1-Me** is by ca. 22 kJ $mol^{-1}$ more stable than ***E*1-Me.** The self-assembly of two ***Z*1-Me** monomers to the H-bonded homo dimer (***Z*1-Me**)$_2$ yields a reaction energy of -187.7 kJ $mol^{-1}$, which translates to a stabilization of 23.5 kJ $mol^{-1}$ per H-bond. Formation of **2-Me** from (***Z*1-Me**)$_2$ is endothermic by 150.7 kJ $mol^{-1}$, which agrees with the observation that **2-Me** is meta-stable and undergoes thermolysis with formation of (***Z*1-Me**)$_2$.

Stereo pictures of the calculated lowest energy conformers of ***Z*1-Me** and ***E*1-Me** as well as (***Z*1-Me**)$_2$ and **2-Me** are depicted in Figures S9a and S9b. The lowest energy conformer of **2-Me** retains the H-bond network of (***Z*1-Me**)$_2$. Calculated distances were visualized with PyMOL.[11]

*Spectral data of* ***Z*1-Me**. UV/Vis (2.0 × $10^{-5}$ mol/L, $CHCl_3$): $\lambda_{max}$ (log $\varepsilon$) 474sh (3.81), 421.5 (4.60), 335.5 (4.31), 322 (4.39), 279 (4.11). CD: (2.5× $10^{-5}$ mol/L, $CHCl_3$) $\lambda_{min/max}$, nm ($\Delta\varepsilon$) 308 (-6.7), 340 (11.7), 480 (3.2). CD: (2.5× $10^{-5}$ mol/L, MeOH) $\lambda_{min/max}$, nm ($\Delta\varepsilon$) 288 (-3.5), 425 (1.1). $^1$H NMR: (please see **Tables S3 and S4** and **Figures S3a and S4a-c**). Fluorescence data (in $CHCl_3$, 6.5 × $10^{-6}$ mol/L): emission ($\lambda_{exc}$ = 420 nm): $\lambda_{max}$ (rel.) 621 (1.0), 485 (0.4) nm; excitation ($\lambda_{em}$ = 621 nm): $\lambda_{max}$ (rel.) 421 (1.0), 318 (0.7). Fluorescence data (in MeOH, 6.0 × $10^{-6}$ mol/L): emission ($\lambda_{exc}$ = 422 nm): $\lambda_{max}$ = 485 nm, excitation (($\lambda_{em}$ = 485 nm): $\lambda_{max}$ (rel.) 421 (1.0), 306 (0.4).

*Single crystals of* ***Z*1-Me**. For recrystallization of ***Z*1-Me**, a solution of 7.5 mg of the crystalline ***Z*1-Me** was dissolved in $CHCl_3$ (2.5 mL). The solution was filtered through a tight plug of cotton wool and the filtrate was collected in a 5 mL vessel. Hexane (2 mL) was slowly added above $CHCl_3$ to form two layers. After ca. 2 weeks, single crystals were obtained.

**X-ray crystal structure analysis.**

X-ray data of a suitable single crystal were collected on the beamline ID29 at the European Synchrtron Radiation Facility (ESRF, Grenoble, France) with wavelength of 75 pm at 100 K. The structure was solved using intrinsic phasing with SHELXTL-XT 2014/4 [9] and refined with SHELXL-2014/7.[10] Hydrogens at nitrogen atoms were found and refined with bond restraints. The absolute structure could be clearly determined about anomalous dispersion of solvent chloroform. Further details, especial disorder problems, are described in the section *_refine_special_details* of the deposited cif-file.

***E*1-Me from photoisomerization of *Z*1-Me**

***Z*1-Me** (7.00 mg, 10.7 µmol) was dissolved in 50 mL Ar-purged of MeOH. The solution was irradiated with the fluorescent lamp under Ar at 0 °C. After 7.5 hours, the isomerisation of ***Z*1-Me** to ***E*1-Me** was rather photostationary (1 : 1) as observed by HPLC analysis at 320 nm. The solvent was evaporated at 0 °C under reduced pressure. The yellow residue was dissolved in MeOH / $H_2O$ (6 mL / 4 mL, v / v) and separated on a reversed phase C-18 column (2 cm × 15 cm) at 6 °C. ***E*1-Me** was washed down by MeOH / $H_2O$ (70 / 30, v / v) as light yellow fraction. The obtained yellow fraction was concentrated under reduced pressure, to remove MeOH, and then lyophilized. ***E*1-Me** was obtained as 1.8 mg as light yellow powder, yield is 26 %. ***Z*1-Me** was recycled by 4.5 mg.

UV/Vis ($2.3\times 10^{-5}$ mol/L, $CHCl_3$) $\lambda_{max}$ (log $\varepsilon$) 425 (4.24), 309 (4.32), UV/Vis ($2.3\times 10^{-5}$ mol/L, MeOH): $\lambda_{max}$ (log $\varepsilon$) 428.5 (4.26), 309.5 (4.36). CD: ($2.3 \times 10^{-5}$ M, MeOH) $\lambda_{min/max}$, ($\Delta\varepsilon$) = 284 (-3.3), 310 (0.8), 425 (1.0). CD: ($2.3 \times 10^{-5}$ M, $CHCl_3$) $\lambda_{min/max}$, ($\Delta\varepsilon$) = 293 (-8.9), 308 (-7.7), 340 (6.0), 426 (1.4), 470 (2.0). Fluorescence data (in MeOH, $1.2 \times 10^{-5}$ mol/L) emission ($\lambda_{exc}$ = 426 nm): $\lambda_{max}$ (rel.) 505 (1.0), excitation ($\lambda_{em}$ = 505 nm): $\lambda_{max}$ (rel.) 390 (1.0),

305 (1.0). $^{1}$H NMR: (**Table S3** and **Figures S6c-d**). ESI-MS found m/z (%) 1353.0 (10), 1352.1 (15), 1351.1 (20, $[2M+K]^+$), 1337.1 (21), 1336.3 (40), 1335.1 (46, $[2M+Na]^+$), 1315.1 (17), 1314.1 (35), 1313.0 (36, $[2M+H]^+$), 696.3 (6), 695.2 (14, $[M+K]^+$), 681.3 (10), 680.3 (31), 679.3 (66, $[M+Na]^+$), 659.2 (9), 658.2 (36), 657.2 (100, $[M+H]^+$ Calcd. for $[M+H]^+$ $C_{36}H_{41}N_4O_8$ *m/z* = 657.3.), 626.3 (8), 625.2 (25, $[M-CH_3OH+H]^+$).

**Preparation of 2-Me by photodimerization of *Z*1-Me**

Crystalline ***Z*1-Me** (3.20 mg, 4.9 µmol) was dissolved in 0.6 mL of $CDCl_3$. The solution was purged with Ar and irradiated with the fluorescent lamp under Ar at 0 °C. After 40 hours, the conversion of ***Z*1-Me** to **2-Me** was complete based on NMR data (purity > 95 %). The solvent was removed under reduced pressure and 3.15 mg (2.4 µmol) of **2-Me** were obtained as a pale yellow residue (98% yield), analysed as follows:

UV/Vis (1.2 × $10^{-5}$ mol/L, $CHCl_3$): $\lambda_{max}$ (log $\varepsilon$) 323 (4.61), 271 (4.53), 240 (4.74). CD (1.25 × $10^{-5}$ mol/L, $CHCl_3$) $\lambda_{min/max}$, nm ($\Delta\varepsilon$) 281 (-24.3), 316 (-16.0), 340 (26.0), 361 (-2.4). $^{1}$H NMR: (**Tables S3** and **S5** and **Figures S8b-d**). ESI-MS m/z (%): 1373.1 (13, $[M-H+Na+K]^+$); 1355.2 (7), 1354.2 (12), 1353.3 (17), 1352.3 (37), 1351.3 (54, $[M+K]^+$); 1339.5 (7), 1338.4 (11), 1337.3 (33), 1336.3 (74), 1335.2 (100, $[M+Na]^+$); 1314.9 (6), 1314.2 (13), 1313.3 (16, $[M+H]^+$, $m/z_{calc}$ ($C_{72}H_{81}N_8O_{16}$) = 1313.6); 696.3 (9), 695.2 (24, $[M-C_{36}H_{40}N_4O_8+K]^+$), 681.5 (6), 680.3 (25), 679.2 (60, $[M-C_{36}H_{40}N_4O_8+Na]^+$), 658.3 (11), 657.2 (24, $[M-C_{36}H_{40}N_4O_8+H]^+$).

**Photochemical experiments with *Z*1-Me**

A solution of ***Z*1-Me** in Ar-purged MeOH (2.5 × $10^{-4}$ M) was irradiated by the fluorescent lamp at 0 °C. HPLC analysis of the solution indicated the formation of ***E*1-Me**. Equilibrium between ***Z*1-Me** and ***E*1-Me** was reached after 6 hours (see **Figure S6a**).

A solution of ***Z*1-Me** in Ar-purged $CHCl_3$ ($1.4 \times 10^{-4}$ M) was irradiated by the fluorescent lamp at 0 °C. HPLC analysis of the solution indicated the clear conversion from ***Z*1-Me** to **2-Me**. (see **Figure S8a**)

**Photochemical experiments with *E*1-Me**

A solution of ***E*1-Me** in Ar-purged MeOH ($3.0 \times 10^{-4}$ M) was irradiated by the fluorescent lamp at 0 °C. HPLC analysis of the solution indicated the formation of ***Z*1-Me**. Equilibrium between ***E*1-Me** and ***Z*1-Me** was reached after 3 hours (see **Figure S6a**).

The solution of ***E*1-Me** (1 mg, $3 \times 10^{-3}$ M) in 0.5 mL Ar-purged $CDCl_3$ was irradiated in a nmr tube by the fluorescent lamp at 0 °C. $^1$H NMR spectra was recorded after certain time (1.5, 3, 6, 10 hours). On basis of NMR analysis, ***E*1-Me** was converted to ***Z*1-Me** first and then **2-Me** was observed in the NMR spectra (see **Figure S7**).

**Photochemical experiments with *Z*1 in $CHCl_3$/dioxane**

***Z*1** was dissolved in Ar-purged $CHCl_3$ / dioxane (99 / 1, v / v, $3.0 \times 10^{-4}$ M) in a 1 cm UV/Vis cell. The absorption and CD spectra (**Figures S5b and S5d**) of ***Z*1** were recorded before irradiation by the fluorescent lamp at 0 °C. After irradiation by 120 minutes, the solution was analysed by HPLC (see **Figure S12a**).

**Photochemical experiments with *Z*1 in 50 mM phosphate buffer (pH 7)**

***Z*1** ($7 \times 10^{-6}$ M) was dissolved in Ar-purged 50 mM $KH_2PO_4$ / $K_2HPO_4$ buffer pH 7 in a 10 cm UV/Vis cell. The absorption spectrum of ***Z*1** was recorded (**Figure S5b**) before irradiation by the fluorescent lamp at 23 °C. Following 160 min irradiation, the solution was analysed by HPLC (see **Figure S10b**). Then, this solution was left in the dark at 23 °C and HPLC was employed to monitor the back isomerisation of ***E*1** to ***Z*1** (see **Figure S10b**).

**Photochemical experiments with *Z*1 in aqueous SDS solution**

***Z*1** (1 mg) was dissolved in Ar-purged 16.4 mM SDS aqueous solution (6 mL). [***Z*1**] = $2.6 \times 10^{-4}$ M, [micelles] = $(16.4 \times 10^{-3} - 8 \times 10^{-3}) / 62 = 1.35 \times 10^{-4}$ M, occupancy rate is roughly two molecules of ***Z*1** in one micelle

The Ar-purged solution (0.2 mL) was added into a 1 mm UV/Vis cell. Then, the absorption spectra of ***Z*1** were recorded during the irradiation by the fluorescent lamp at 23 °C (**Figure S11a**). After irradiation by 180 minutes, the solution was analysed by HPLC (see **Figure S11b**).

**Thermolytic cleavage of 2-Me to *Z*1-Me**

A stock solution ($1.65 \times 10^{-4}$ mol/L) of **Z1-Me** in acid free $CHCl_3$ was prepared. A 1 mm UV/Vis cell was filled with 0.3 mL of the stock solution and purged with Ar. The solution was then irradiated at 0 °C by the fluorescent lamp for 120 minutes, until no further absorption at 420 nm decreases in the absorption spectra. Subsequently, the solution of **2-Me** was left at 23 °C in darkness for certain time. Absorption spectroscopy was employed to monitor the decomposition reaction. Four parallel experiments were also performed to monitor the decomposition reaction of **2-Me** at 5 °C, 30 °C, 40 °C, and 50 °C, respectively. The absorption at 420 nm was obtained in order to analyze the decomposition kinetics (**Figure S13**).

*Acid accelerated cleavage of* ***2-Me to Z1-Me***

To the solution **2-Me** (2.8 mL, $7.0 \times 10^{-5}$ M, obtained from 6.5 hours irradiation of **Z1-Me** in Ar-purged acid free $CHCl_3$) was added TFA (5 µL) at 23 °C. HPLC was employed to monitor the decomposition process. (See **Figure S8a**)

**Decomposition of 2 in aqueous SDS solution**

After formation of **2** by irradiation of an aqueous SDS solution of ***Z*1** for 7.5 hours, the aqueous SDS solution was left to decompose in the dark. HPLC was used to monitor the decomposition process (see **Figure S11c**)

**Isomerization reaction of *E*1-Me**

The solution of ***E*1-Me** (2.0 × $10^{-4}$ M) in 0.5 mL of Ar-purged $CHCl_3$ was treated with 5µL of AcOH at 23 °C. ***E*1-Me** converted to ***Z*1-Me** in the course of 5 hours, as analysed by HPLC (see **Figure S6b**).

**Table S1.** Crystal data and structure refinement for ***Z*1-Me**.

| | | |
|---|---|---|
| Empirical formula | $C_{36}H_{40}N_4O_8 \times CHCl_3$ | |
| Formula weight | 756.09 | |
| Temperature | 100(2) K | |
| Wavelength | 0.75 Å | |
| Crystal system | Orthorhombic | |
| Space group | P $2_12_12_1$ | |
| Unit cell dimensions | a = 8.926(1) Å | α = 90°. |
| | b = 17.516(2) Å | β = 90°. |
| | c = 49.230(6) Å | γ = 90°. |
| Volume | 7697.0(15) $Å^3$ | |
| Z | 8 | |
| Density (calculated) | 1.339 $g/cm^3$ | |
| Absorption coefficient | 0.293 $mm^{-1}$ | |
| F(000) | 3248 | |
| Crystal size | 0.21 × 0.13 × 0.11 $mm^3$ | |
| Theta range for data collection | 1.746 to 22.739° | |
| Index ranges | -9<=h<=9, -17<=k<=17, -50<=l<=50 | |
| Reflections collected | 8448 | |
| Independent reflections | 4396 [R(int) = 0.0509] | |
| Reflections [I>2sigma(I)] | 7334 | |
| Completeness to theta = 21.49° | 98.0 % | |
| Refinement method | Full-matrix least-squares on $F^2$ | |
| Data / restraints / parameters | 8448 / 25 / 1094 | |
| Goodness-of-fit on $F^2$ | 1.025 | |
| Final R indices [I>2sigma(I)] | R1 = 0.0796, wR2 = 0.2187 | |
| R indices (all data) | R1 = 0.0899, wR2 = 0.2347 | |
| Absolute structure parameter | -0.02(4) | |
| Largest diff. peak and hole | 0.600 and -0.228 e.$Å^{-3}$ | |

**Table S2.** Hydrogen bonds observed in the crystal structure of ***Z*1-Me**.

| D-H...A | d(D-H) / Å | d(H...A) / Å | d(D...A) / Å | <(DHA) / ° |
|---|---|---|---|---|
| N(21)-H...O(591) | 0.89(9) | 1.97(9) | 2.830(10) | 160(8) |
| N(22)-H...O(591) | 0.89(7) | 1.87(8) | 2.739(9) | 165(8) |
| N(23)-H...O(601) | 0.90(7) | 1.97(7) | 2.818 (9) | 157(8) |
| N(24)-H...O(601) | 0.88(6) | 1.92(6) | 2.744(10) | 165(7) |
| N(61)-H...O(191) | 0.86(7) | 2.03(7) | 2.849(9) | 159(8) |
| N(62)-H...O(191) | 0.88(7) | 1.96(6) | 2.783(9) | 156(8) |
| N(63)-H...O(201) | 0.87(8) | 1.98(8) | 2.845(11) | 169(8) |
| N(64)-H...O(201) | 0.89(8) | 1.91(8) | 2.779(10) | 168(8) |

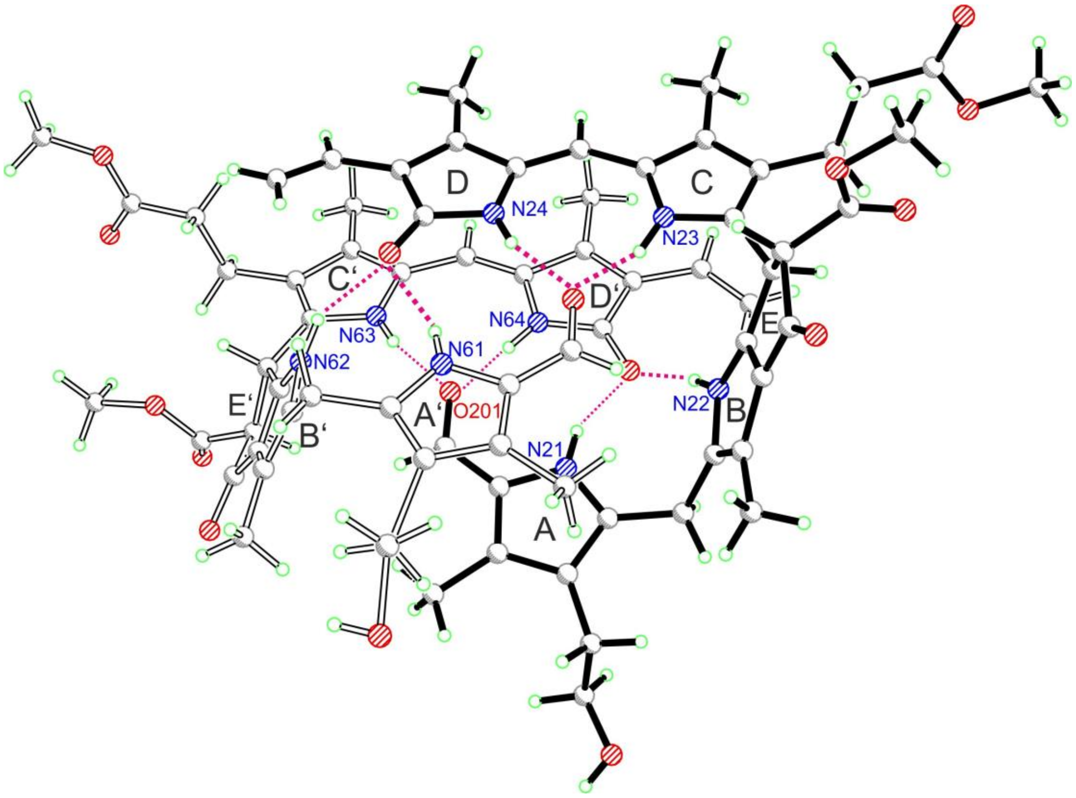


**Figure S1.** Structure of the non-covalent homodimer (***Z*1-Me**)$_2$, highlighting H-bonds between two ***Z*1-Me** modules.

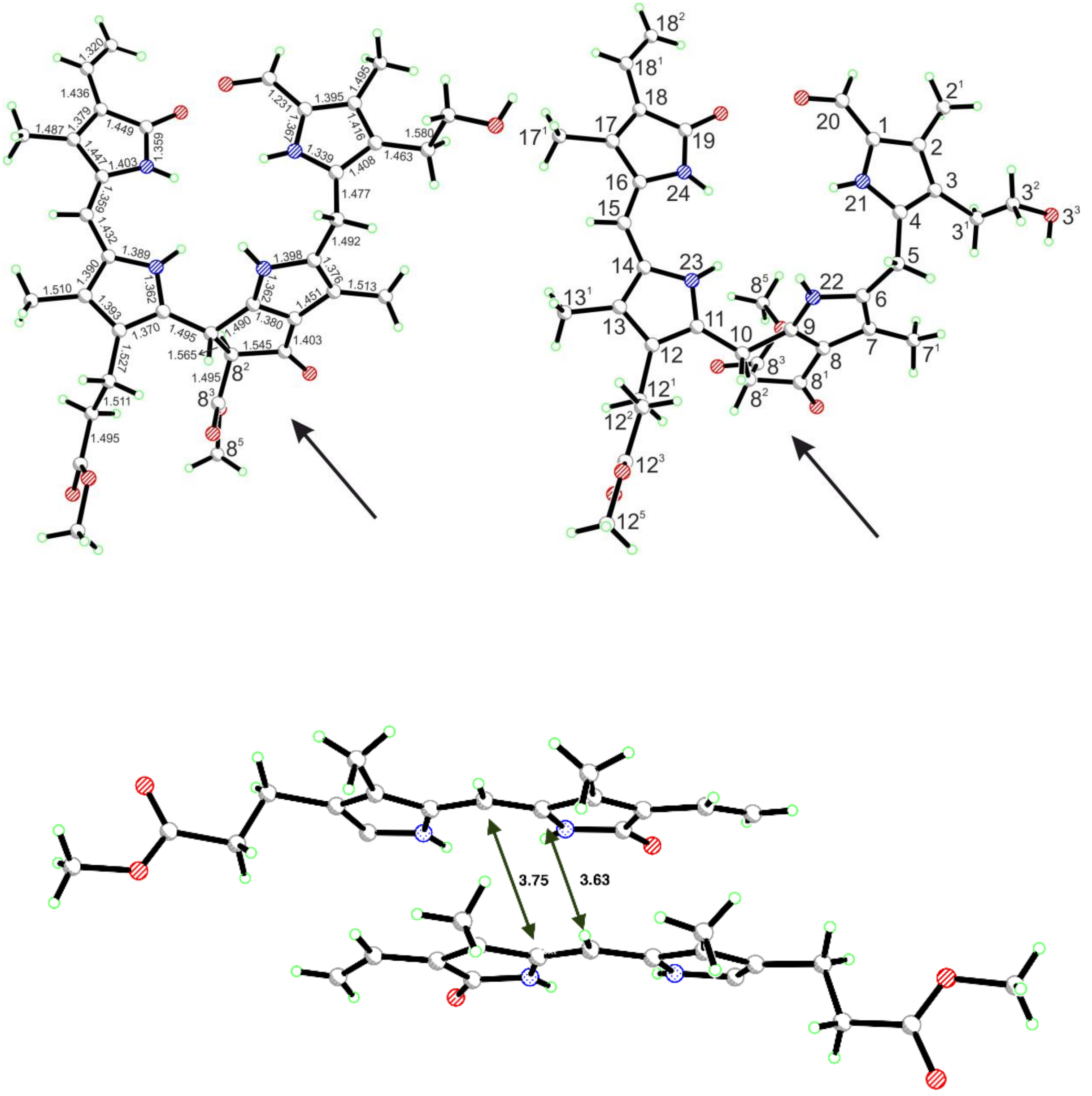


**Figure S2.** Top: Ball and stick model of the crystal structure of ***Z*1-Me** and numerical values for bond lengths (left) and of the $8^2$-epimer ***epi*-*Z*1-Me**, depicting atom numbering (right). Bottom: Structures of the C/D-moieties of ***Z*1-Me** in the non-covalent dimer structure (***Z*1-Me**)$_2$ and distances between the carbons atoms (C15-C16’ and C15’-C16), between which the new bonds are formed in the [2+2]-cycloaddition reaction (other parts of (***Z*1-Me**)$_2$ were deleted for clarity).

**Table S3** $^{1}$H- and $^{13}$C-NMR signal assignments of the main isomers of ***Z*1-Me**, ***E*1-Me** and **2-Me** in $CDCl_3$.

| | ***Z*1-Me** $^{1}$H [a] | ***Z*1-Me** $^{13}$C | ***E*1-Me** $^{1}$H [a] | ***E*1-Me** $^{13}$C | **2-Me** $^{1}$H [a] | **2-Me** $^{13}$C |
|---|---|---|---|---|---|---|
| C-1 | | 127.9 | | 128.0 | | 128.5 |
| C-2 | | 135.7 | | 136.2 | | 136.7 |
| $H_3C$-2$^1$ | 1.80 (s) | 9.0 | 2.14 (s) | 8.9 | 2.28 (s) | 9.1 |
| C-3 | | 118.0 | | 120.2 | | 120.8 |
| $H_2C$-3$^1$ | 2.67 / 2.86 (m) | 27.2 | 2.56 (m) | 26.8 | 2.49 / 2.59 (m) | 27.0 |
| $H_2C$-3$^2$ | 3.58 / 3.69 (m) | 62.6 | 3.66 /3.69 (m) | 61.9 | 3.45 / 3.51 (m) | 61.4 |
| HO-3$^3$ | 1.65 (br.s) | | 1.65 (br.s) | | | |
| C-4 | | 139.3 | | 141.1 | | 142.2 |
| $H_2C$-5 | 3.79 / 4.06 (AB, 15.9 Hz) | 22.1 | 3.85 / 3.94 (AB, 18 Hz) | 22.0 | 3.71 / 4.08 ( AB, 19.1 Hz) | 22.9 |
| C-6 | | 133.3 | | 131.2 | | 130.7 |
| C-7 | | 110.8 | | 113.2 | | 113.4 |
| $H_3C$-7$^1$ | 2.26 (s) | 10.0 | 2.03 (s) | 9.0 | 1.96 (s) | 8.8 |
| C-8 | | 125.2 | | 126.1 | | 125.1 |
| C-8$^1$ | | 188.6 | | 188.2 | | 187.8 |
| HC-8$^2$ | 3.80 (d, 5.3 Hz) | 66.5 | 3.58 (d, 1.5 Hz) | 69.1 | 3.74 (d, 3.7 Hz) | 67.1 |
| C-8$^3$ | | 169.6 | | 169.2 | | 169.4 |
| $H_3C$-8$^5$ | 3.79 (s) | 52.6 | 3.82 (s) | 52.9 | 3.77 (s) | 52.7 |
| C-9 | | 155.4 | | 157.2 | | 158.6 |
| HC-10 | 5.15 (d, 5.3 Hz) | 36.5 | 5.18 (d, 1.5 Hz) | 35.6 | 5.05 (d, 3.7 Hz) | 35.1 |
| C-11 | | 130.5 | | 131.5 | | 125.6 |
| C-12 | | 122.6 | | 120.9 | | 118.6 |
| $H_2C$-12$^1$ | 2.69 / 2.75 (m) | 19.4 | 2.85 (m) | 19.3 | 2.65 / 2.75 (m) | 19.4 |
| $H_2C$-12$^2$ | 2.31 / 2.46 (m) | 35.0 | 2.55 (m) | 35.0 | 2.26 / 2.45 (m) | 36.3 |
| C-12$^3$ | | 173.4 | | 173.5 | | 173.8 |
| $H_3C$-12$^5$ | 3.72 (s) | 51.7 | 3.66 (s) | 51.7 | 3.44 (s) | 51.7 |
| C-13 | | 126.4 | | 123.8 | | 117.6 |
| $H_3C$-13$^1$ | 2.13 (s) | 9.9 | 2.08 (s) | 10.0 | 1.74 (s) | 9.1 |
| C-14 | | 124.3 | | 120.9 | | 118.6 |
| HC-15 | 5.96 (s) | 101.7 | 5.89 (s) | 107.6 | 4.33 (s) | 43.7 |
| C-16 | | 127.8 | | 134.8 | | 73.1 |
| C-17 | | 141.6 | | 137.5 | | 150.4 |
| $H_3C$-17$^1$ | 2.12 (s) | 9.9 | 1.74 (s) | 12.4 | 2.22 (s) | 10.9 |
| C-18 | | 122.6 | | 128.3 | | 128.4 |
| HC-18$^1$ | 5.97 (dd, 11.6 / 17.7 Hz ) | 125.8 | 6.02 (dd, 11.6 / 17.6 Hz) | 125.0 | 6.25 (dd, 11.6 / 17.6 Hz) | 124.9 |
| $H_2C$-18$^2$ | 5.10 (dd, 1.2 / 11.6 Hz)<br>5.75 (dd, 1.2 / 17.7 Hz) | 116.5 | 5.19 (dd, 1.3 / 11.6 Hz)<br>5.88 (dd, 1.3 / 17.6 Hz) | 119.4 | 5.38 (dd, 1.3 / 11.6 Hz)<br>6.06 (dd, 1.3 / 17.6 Hz) | 121.1 |
| C-19 | | 171.6 | | 170.1 | | 172.3 |
| HC-20 | 7.24 (s) | 176.1 | 8.75 (s) | 175.1 | 8.68 (s) | 176.4 |
| HN21 | 12.29 (s) | | 9.12 (s) | | 9.83 (s) | |
| HN22 | 11.49 (s) | | 10.78 (s) | | 11.04 (s) | |
| HN23 | 10.90 (s) | | 9.58 (s) | | 10.61 (s) | |
| HN24 | 11.16 (s) | | 5.71 (s) | | 6.66 (s) | |

[a] chemical shift (signal type, coupling constant)

**Table S4** $^{1}$H- and $^{13}$C-NMR signal assignments of non-covalent dimers (***Z*1-Me / *Z*1-Me**) and (***Z*1-Me / *epi*-*Z*1-Me**) in $CDCl_3$.

| | ***Z*1-Me / *Z*1-Me** [a] | ***Z*1-Me / *Z*1-Me** | ***Z*1-Me / *epi*-*Z*1-Me** | | ***Z*1-Me / *epi*-*Z*1-Me** | |
|---|---|---|---|---|---|---|
| | ***Z*1-Me** $^{1}$H | ***Z*1-Me** $^{13}$C | ***Z*1-Me** $^{1}$H [a] | ***epi*-*Z*1-Me** $^{1}$H [a] | ***Z*1-Me** $^{13}$C | ***epi*-*Z*1-Me** $^{13}$C |
| C-1 | | 127.9 | | | 128.6 | 127.9 |
| C-2 | | 135.7 | | | 135.2 | 135.4 |
| $H_3C$-2$^1$ | 1.80 (s) | 9.0 | 2.11 (s) | 1.81 (s) | 9.3 | 9.0 |
| C-3 | | 118.0 | | | 117.9 | 118.0 |
| $H_2C$-3$^1$ | 2.67 / 2.86 (m) | 27.2 | | | | |
| $H_2C$-3$^2$ | 3.58 / 3.69 (m) | 62.6 | | | | |
| HO-3$^3$ | 1.65 (br.s) | | | | | |
| C-4 | | 139.3 | | | | |
| $H_2C$-5 | 3.79 / 4.06 (AB, 15.9 Hz) | 22.1 | | | | |
| C-6 | | 133.3 | | | 133.1 | 133.2 |
| C-7 | | 110.8 | | | 110.8 | 111.0 |
| $H_3C$-7$^1$ | 2.26 (s) | 10.0 | 2.27 (s) | 2.31 (s) | 10.1 | 10.1 |
| C-8 | | 125.2 | | | 125.0 | 127.6 |
| C-8$^1$ | | 188.6 | | | 188.6 | 189.5 |
| HC-8$^2$ | 3.80 (d, 5.3 Hz) | 66.5 | 3.86(d, 5.3 Hz) | 4.08(d, 7.4 Hz) | 66.6 | 65.2 |
| C-8$^3$ | | 169.6 | | | 169.6 | 168.6 |
| $H_3C$-8$^5$ | 3.79 (s) | 52.6 | | 3.15 (s) | | 51.9 |
| C-9 | | 155.4 | | | | 155.8 |
| HC-10 | 5.15 (d, 5.3 Hz) | 36.5 | 5.18(d, 5.3 Hz) | 5.01(d, 7.4 Hz) | 36.5 | 36.2 |
| C-11 | | 130.5 | | | 130.4 | 129.0 |
| C-12 | | 122.6 | | | 122.7 | 122.8 |
| $H_2C$-12$^1$ | 2.69 / 2.75(m) | 19.4 | | | | |
| $H_2C$-12$^2$ | 2.31 / 2.46 (m) | 35.0 | | | | |
| C-12$^3$ | | 173.4 | | | | |
| $H_3C$-12$^5$ | 3.72 (s) | 51.7 | | | | |
| C-13 | | 126.4 | | | 126.5 | 125.9 |
| $H_3C$-13$^1$ | 2.13 (s) | 9.9 | | | | |
| C-14 | | 124.3 | | | 124.3 | 123.8 |
| HC-15 | 5.96 (s) | 101.7 | | | | |
| C-16 | | 127.8 | | | 127.7 | 127.5 |
| C-17 | | 141.6 | | | 141.7 | 141.2 |
| $H_3C$-17$^1$ | 2.12 (s) | 9.9 | | | | |
| C-18 | | 122.6 | | | 122.6 | 122.8 |
| HC-18$^1$ | 5.97 (dd, 11.6 / 17.7 Hz ) | 125.8 | 5.90 (dd, 9.7 / 15.0 Hz) | 6.00 (dd, 9.7 / 15.0 Hz) | 125.8 | 125.8 |
| $H_2C$-18$^2$ | 5.10 (dd, 1.2 / 11.6 Hz)<br>5.75 (dd, 1.2 / 17.7 Hz) | 116.5 | 5.01 (10 Hz)<br>5.75 (15.0 Hz) | 5.09 (10 Hz)<br>5.69 (15.0 Hz) | 116.1 | 116.4 |
| C-19 | | 171.6 | | | | |
| HC-20 | 7.24 (s) | 176.1 | 7.25 (s) | 7.30 (s) | | |
| HN21 | 12.29 (s) | | 12.14 (s) | 12.38 (s) | | |
| HN22 | 11.49 (s) | | 11.57 (s) | 11.68 (s) | | |
| HN23 | 10.90 (s) | | 11.02 (s) | 11.00 (s) | | |
| HN24 | 11.16 (s) | | 11.25 (s) | 11.35 (s) | | |

[a] chemical shift (signal type, coupling constant)

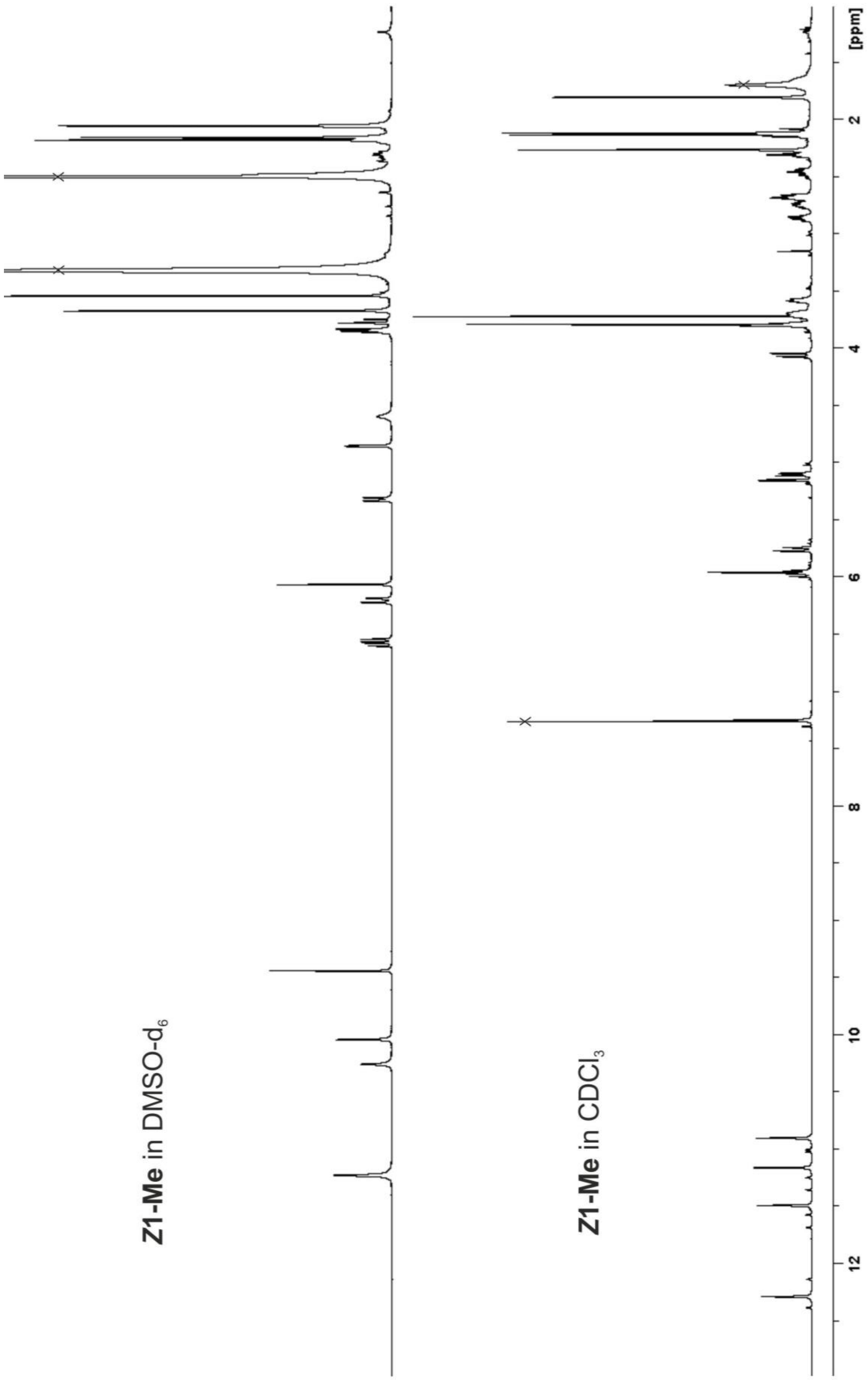


**Figure S3a.** $^{1}$H-NMR spectra of ***Z*1-Me** in DMSO-$d_6$ ($6.1 \times 10^{-3}$ M, 500 MHz, 25°C ) and in $CDCl_3$ ($1.0 \times 10^{-2}$ M, 600 MHz, 2°C); x marks solvent signals.

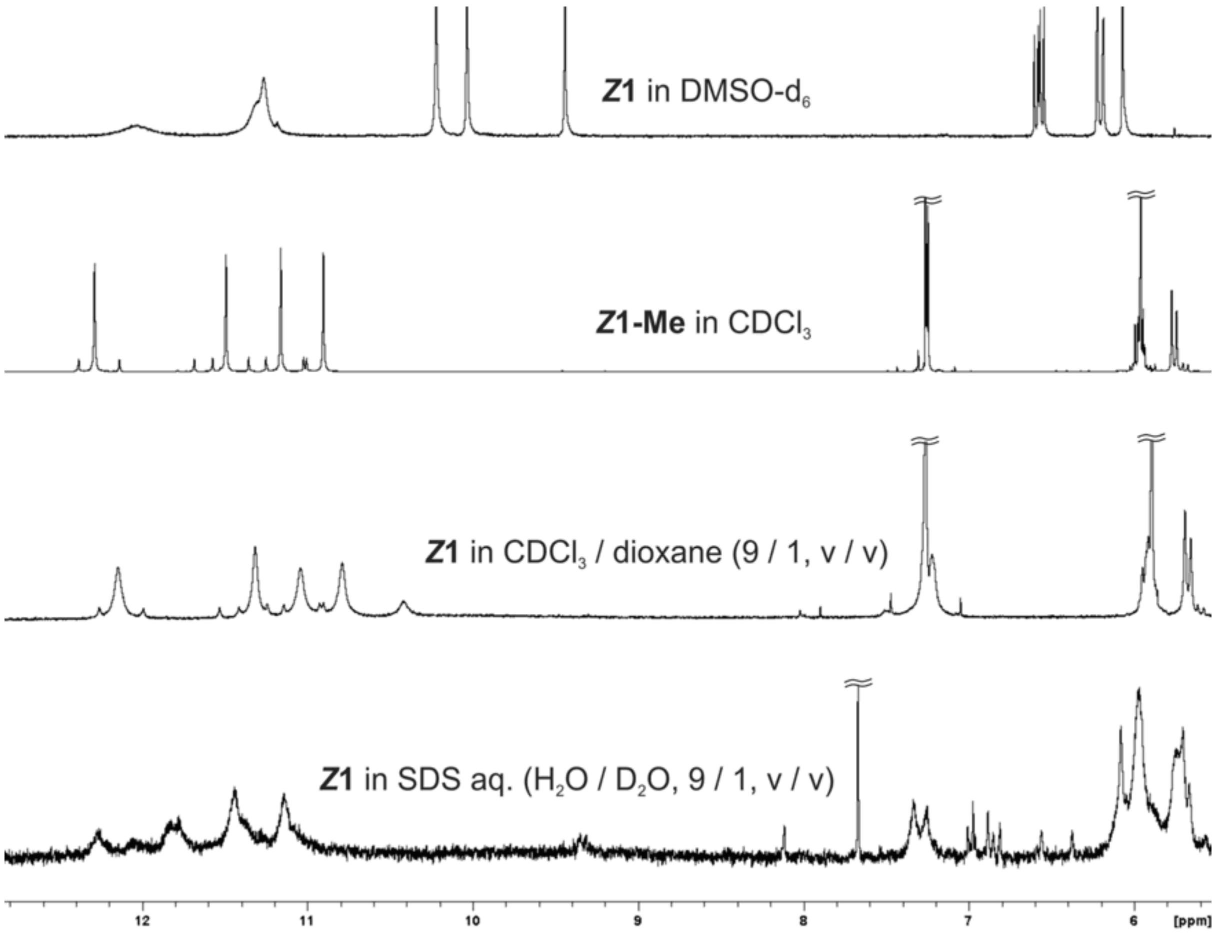


**Figure S3b.** Low field sections of (top) 500 MHz $^1$H NMR spectra of ***Z*1** in DMSO-$d_6$ (4.7 × $10^{-3}$ M, 25°C), (middle) of ***Z*1-Me** in $CDCl_3$ (1.0 × $10^{-2}$ M, 25°C) and (bottom) of ***Z*1** in $CDCl_3$ / dioxane (1.6 × $10^{-3}$ M, 25°C) and in 14.8 mM SDS in $D_2O/H_2O$ (ca. 1.0 × $10^{-4}$ M, 25 °C). The predominant solution structure of ***Z*1-Me** in $CDCl_3$ was indicated as non-covalent H-bonded dimer **(*Z*1-Me)$_2$**. Likewise, on the basis of the similar chemical shift pattern (in the lower field section of the spectra), ***Z*1** in $CDCl_3$ / dioxane and in SDS were also indicated to be the non-covalent H-bonded dimer (***Z*1**)$_2$. In contrast, ***Z*1** in DMSO-$d_6$ is predominantly monomeric (top).

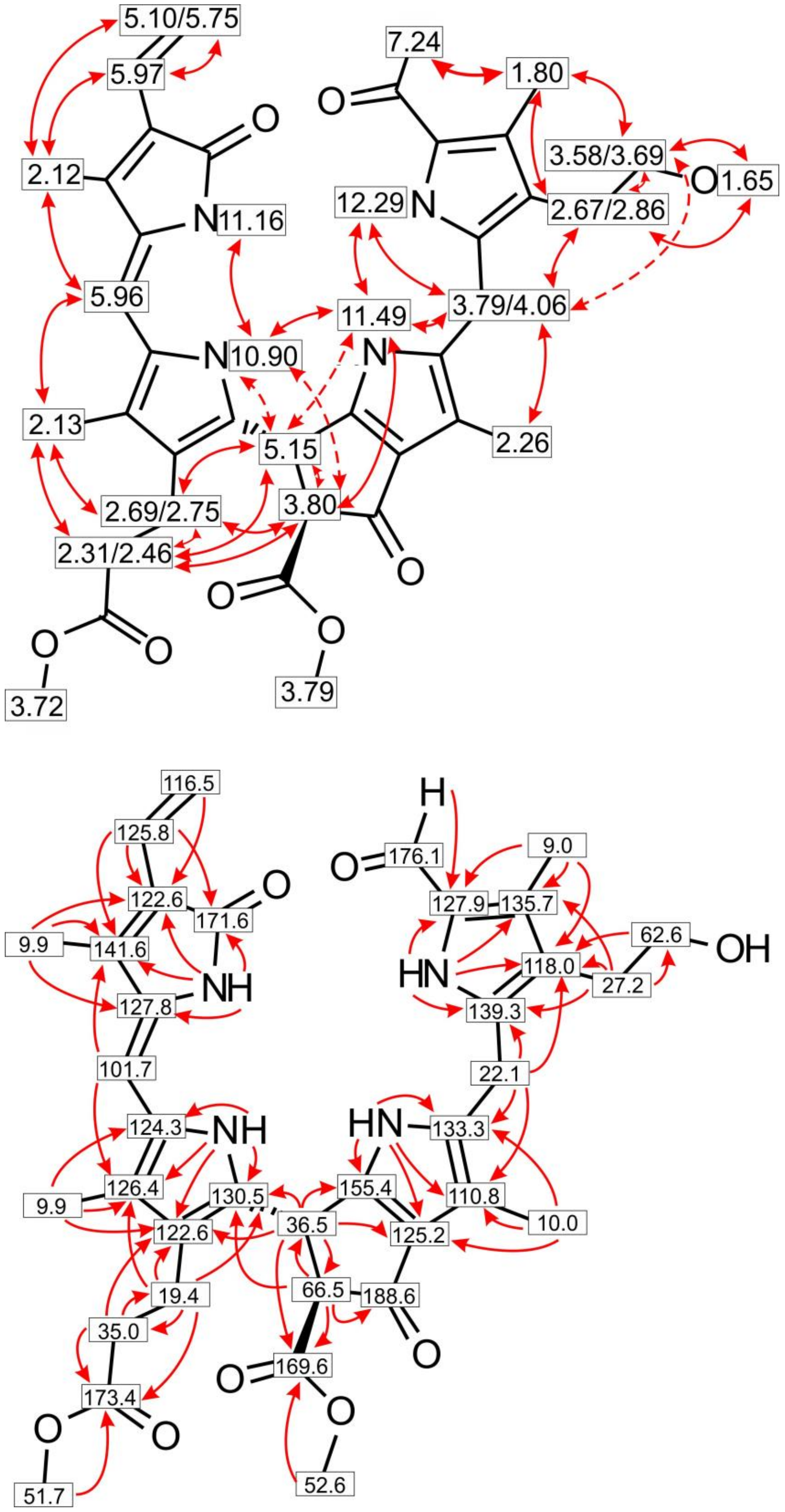


**Figure S4a.** NMR analysis of (***Z*1-Me**)$_2$ in $CDCl_3$ (600 MHz, 2°C). Top: Intramodular $^1H,^1H$-correlations from ROESY spectra. Bottom: $^1H,^{13}C$-heteronuclear correlations from HSQC and HMBC spectra.

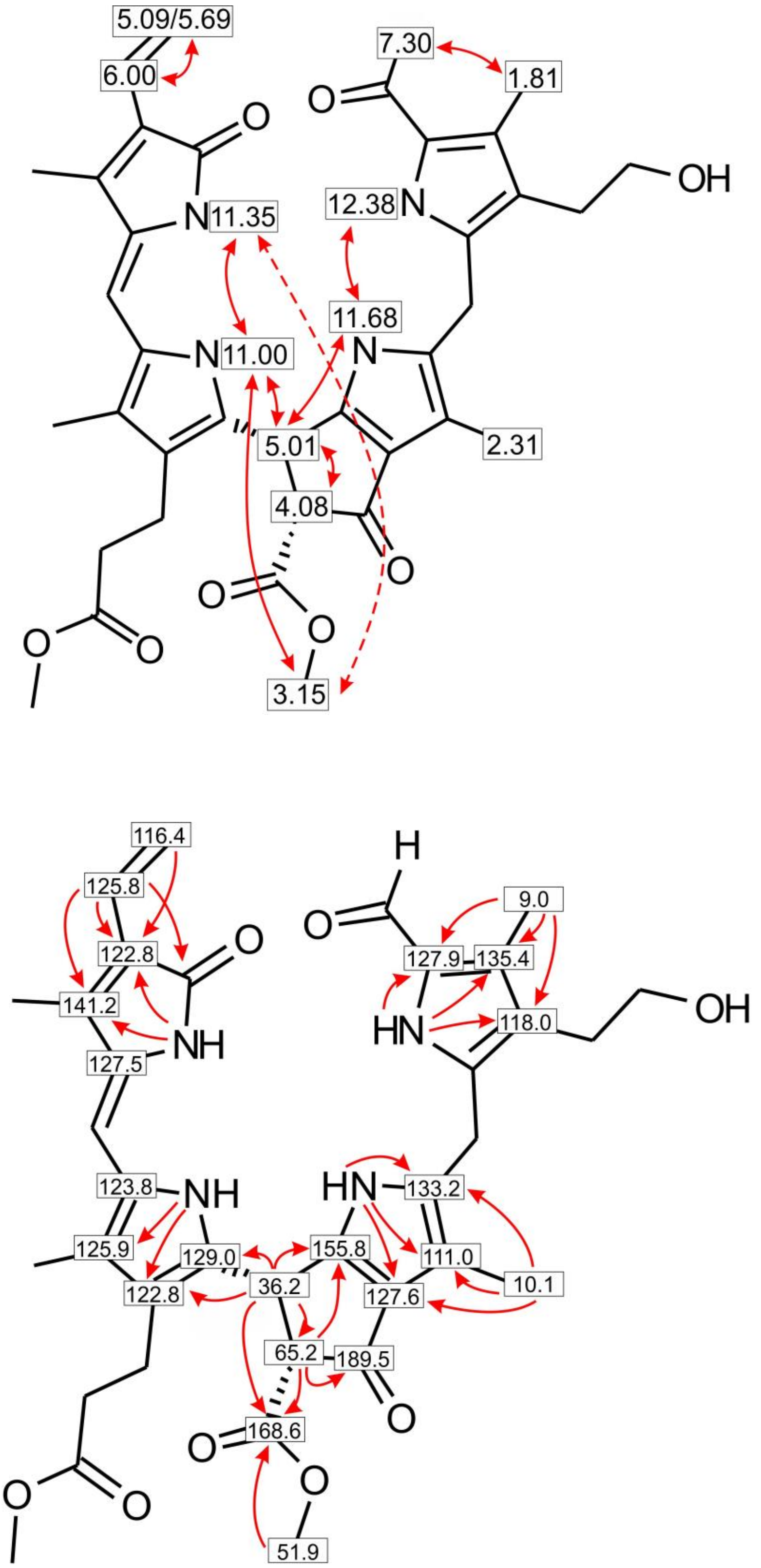


**Figure S4b.** NMR analysis of the ***epi*-*Z*1-Me** moiety of ***Z*1-Me/*epi*-*Z*1-Me** in $CDCl_3$ (600 MHz, 2°C). Top: Intramodular correlations from $^{1}H,^{1}H$ ROESY spectra. Bottom: Heteronuclear correlations from $^{1}H,^{13}C$-HSQC and $^{1}H,^{13}C$-HMBC spectra.

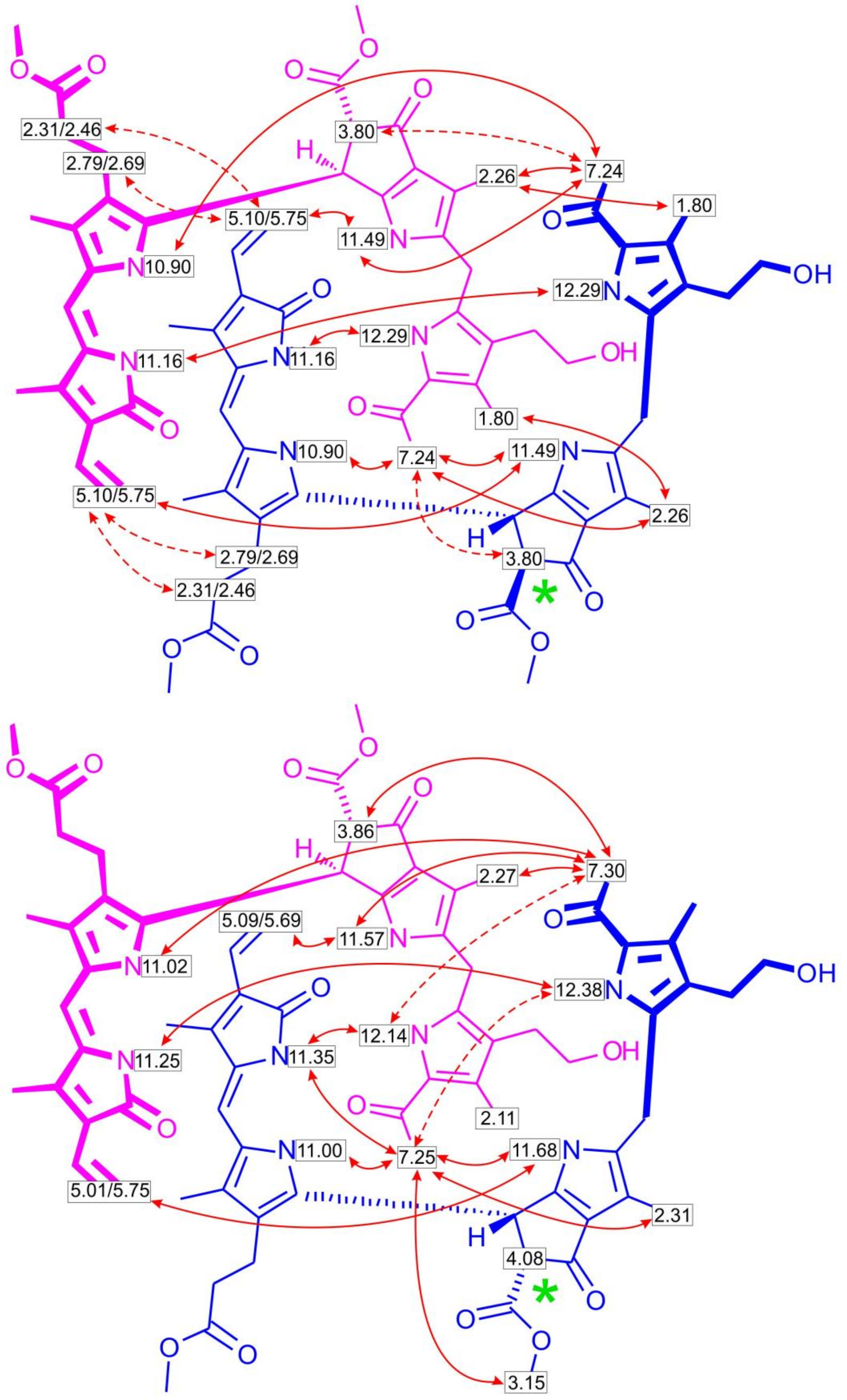


**Figure S4c.** Intermodular homonuclear $^{1}H,^{1}H$-correlations from ROESY spectra in $CDCl_3$ (600 MHz, 2°C). Top: H-bonded homodimer (***Z*1-Me)$_2$**. Bottom: H-bonded heterodimer (***Z*1-Me/*epi*-*Z*1-Me**). The epimerization site is marked with a green star.

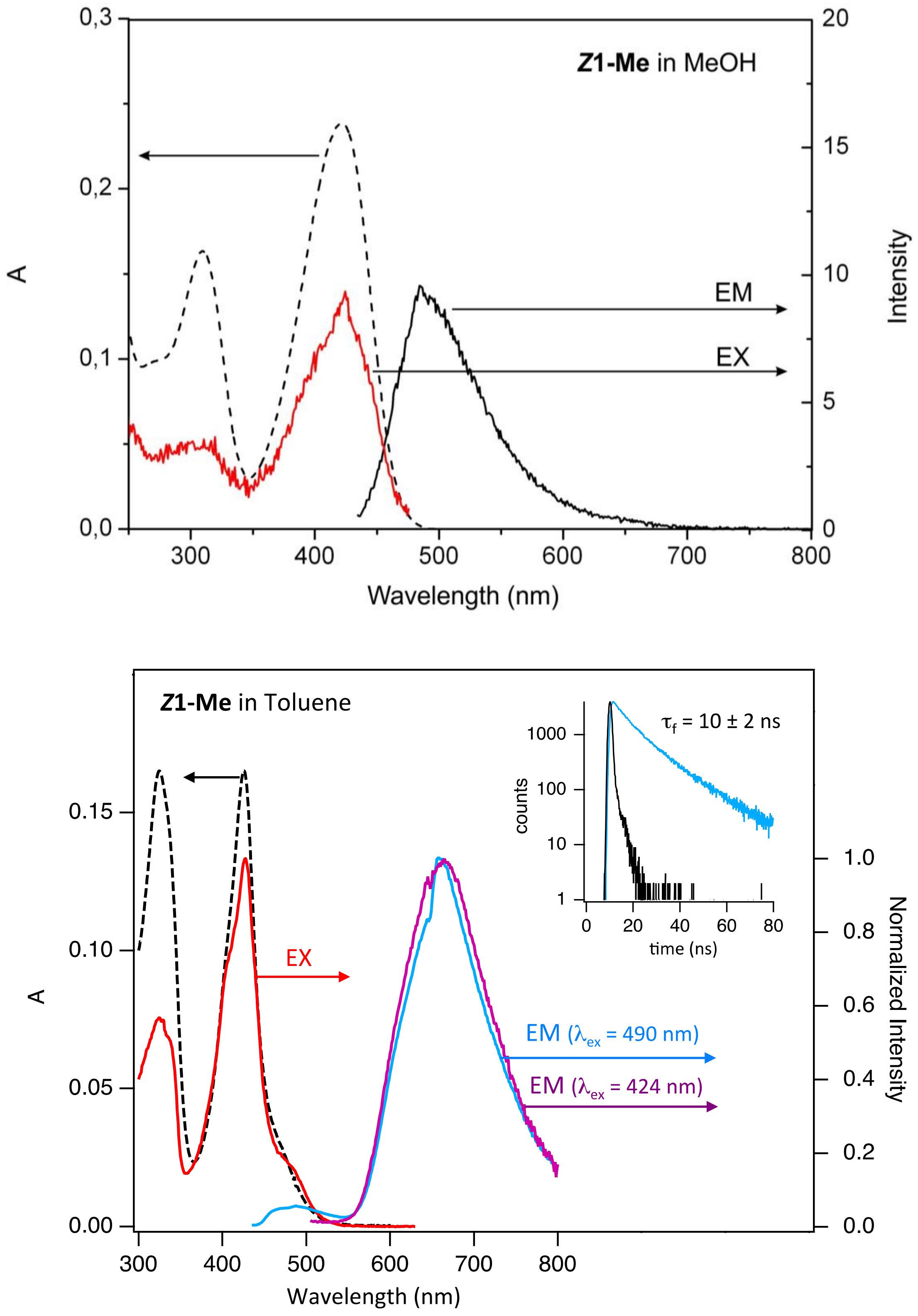


**Figure S5a.** UV/Vis- (dashed line), fluorescence emission (EM) and excitation (EX) spectra (solid lines) spectrum of ***Z*1-Me.** Top: ***Z*1-Me** ($6.0 \times 10^{-6}$ M) dissolved in MeOH, at room temperature. Bottom: UV/Vis-spectrum (room temperature, dashed line) of a solution of ***Z*1-Me** ($4.2 \times 10^{-5}$ M) in toluene, fluorescence emission (EM) and excitation (EX) spectra (solid lines, at 77 K). Inset: Fluorescence decay trace at 77 K monitored at 660 nm (blue) and instruments response function (black).

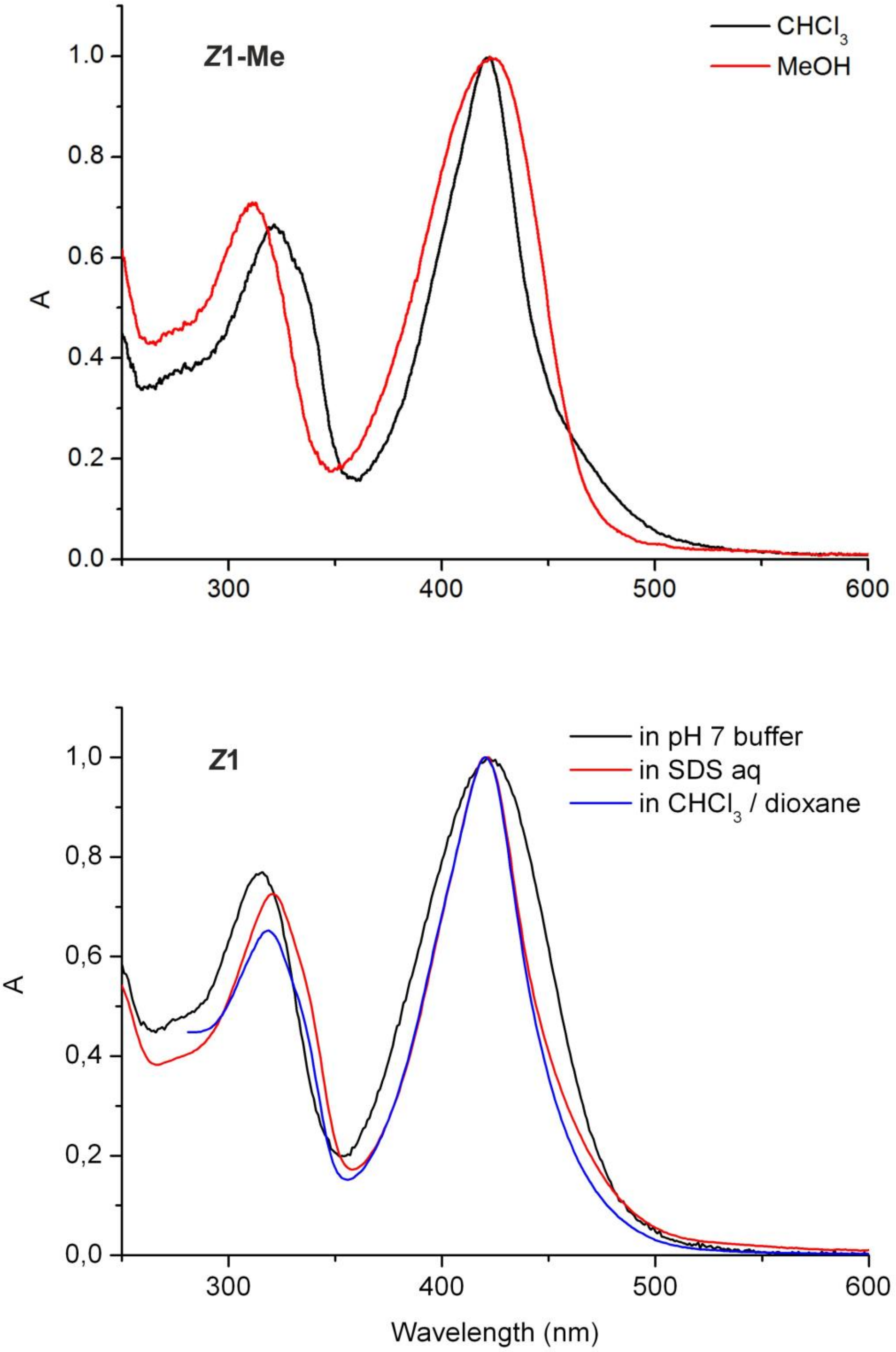


**Figure S5b.** Top: UV/Vis spectra of ***Z*1-Me** in $CHCl_3$ and MeOH (normalized at maxima near 425 nm), indicating ***Z*1-Me** to occur as a non-covalent H-bonded dimer and monomer, respectively. Bottom: UV/Vis spectra of ***Z*1** in different solvents (normalized at maxima near 425 nm). By comparison of the spectra of ***Z*1-Me** and ***Z*1** in different solvents, in aqueous SDS solution (red line) and in $CHCl_3$ / dioxane (blue line) ***Z*1** exists as H-bonded dimer and ***Z*1** is largely monomeric in aqueous buffer at pH 7 (black line).

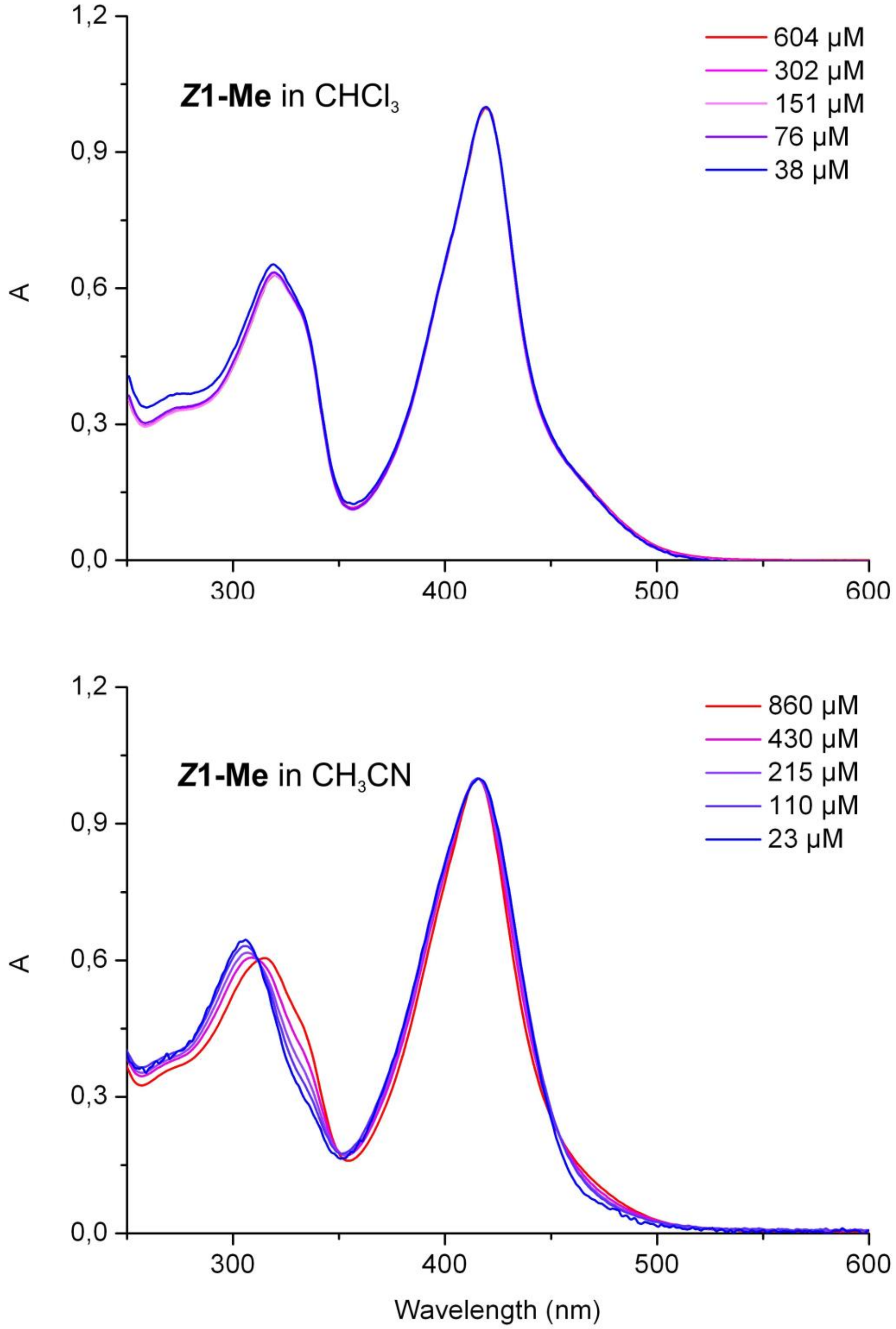


**Figure S5c.** Top: UV/Vis spectra of ***Z*1-Me** in $CHCl_3$ at the indicated concentrations (spectra normalized at 419 nm). Bottom: UV/Vis spectra of ***Z*1-Me** in $CH_3CN$ at different concentrations (spectra. normalized at 415 nm).

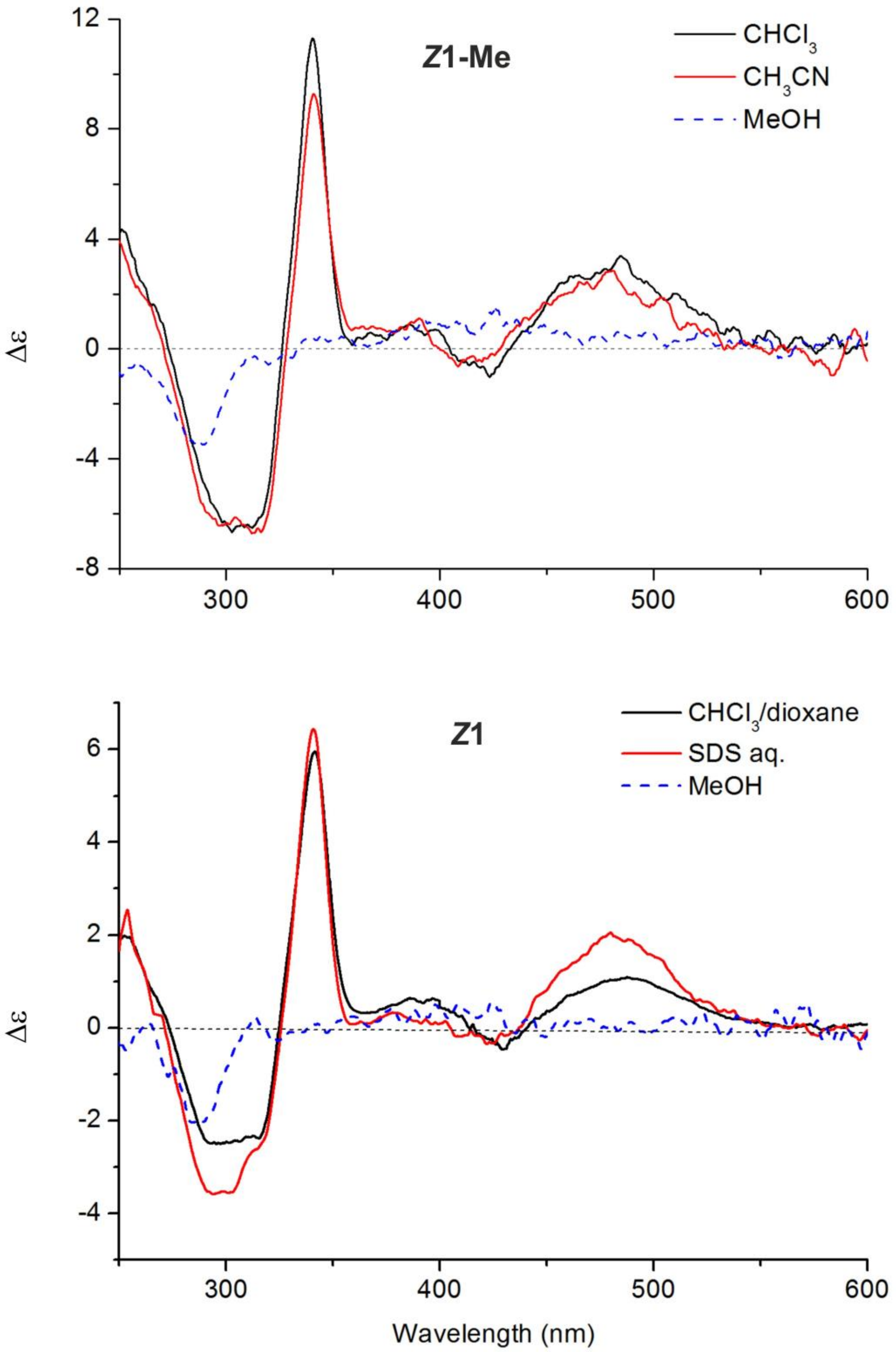


**Figure S5d.** CD spectra of ***Z*1-Me** (top) and of ***Z*1** (bottom) in different solvents at room temperature. The distinct broad absorption around 475 nm and the sharp absorption near 340 nm are attributed to the formation of non-covalent H-bonded dimer, e.g., of ***Z*1-Me** in $CHCl_3$ (top, black line) or of ***Z*1** in SDS aqueous solution and in $CHCl_3$/dioxane (bottom, red and black lines, resp.).

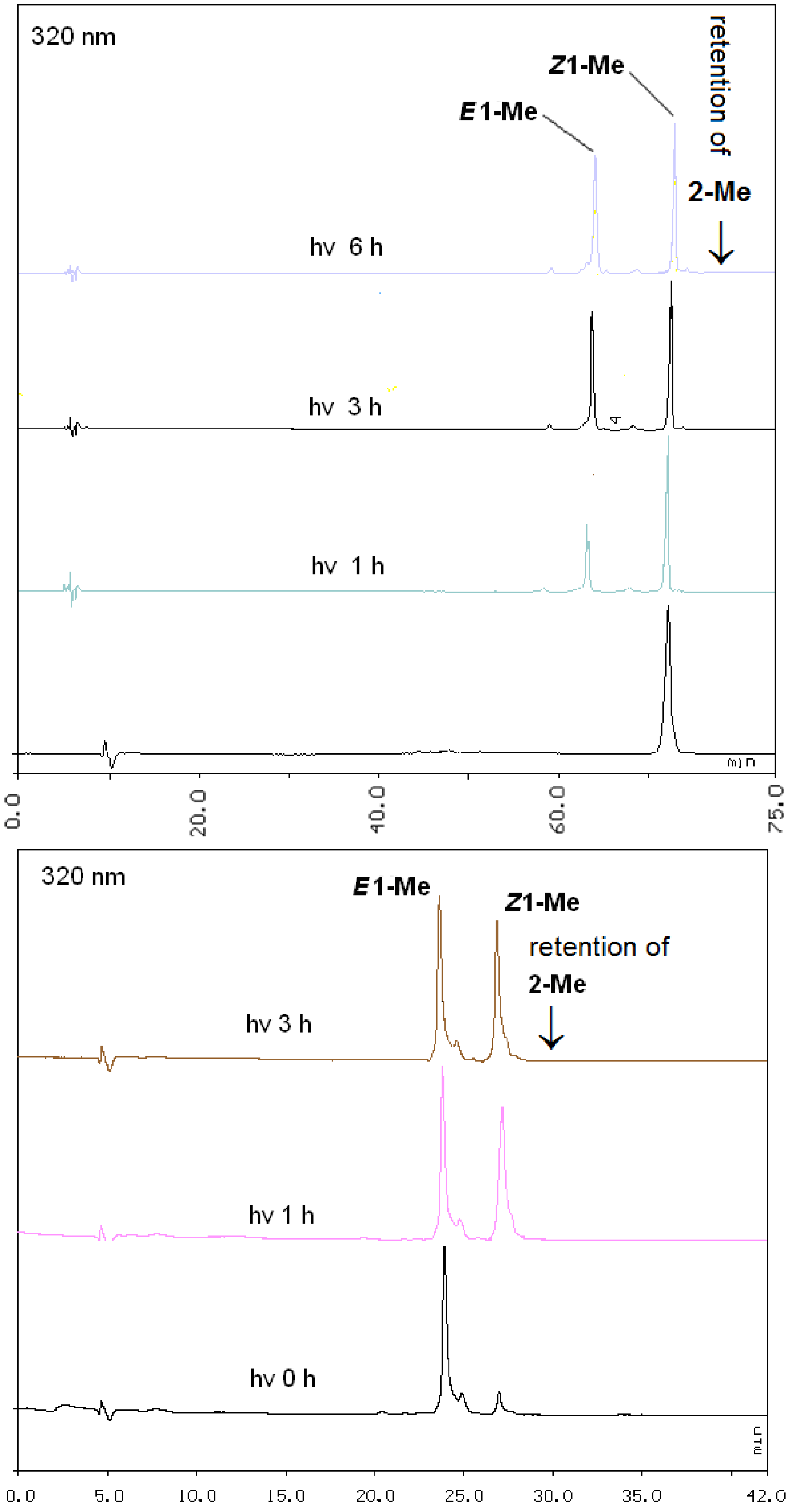


**Figure S6a.** E/Z-photoisomerization of ***Z*1-Me** and ***E*1-Me**. Top: HPLC-analysis (Sosy I) of a solution of ***Z*1-Me** (2.5 × $10^{-4}$ M) in MeOH before and after irradiation by the fluorescent lamp at 0°C, Bottom: HPLC-analysis (Sosy II) of a solution of ***E*1-Me** (3.0 × $10^{-4}$ M) in MeOH before and after irradiation by the fluorescent lamp at 0°C.

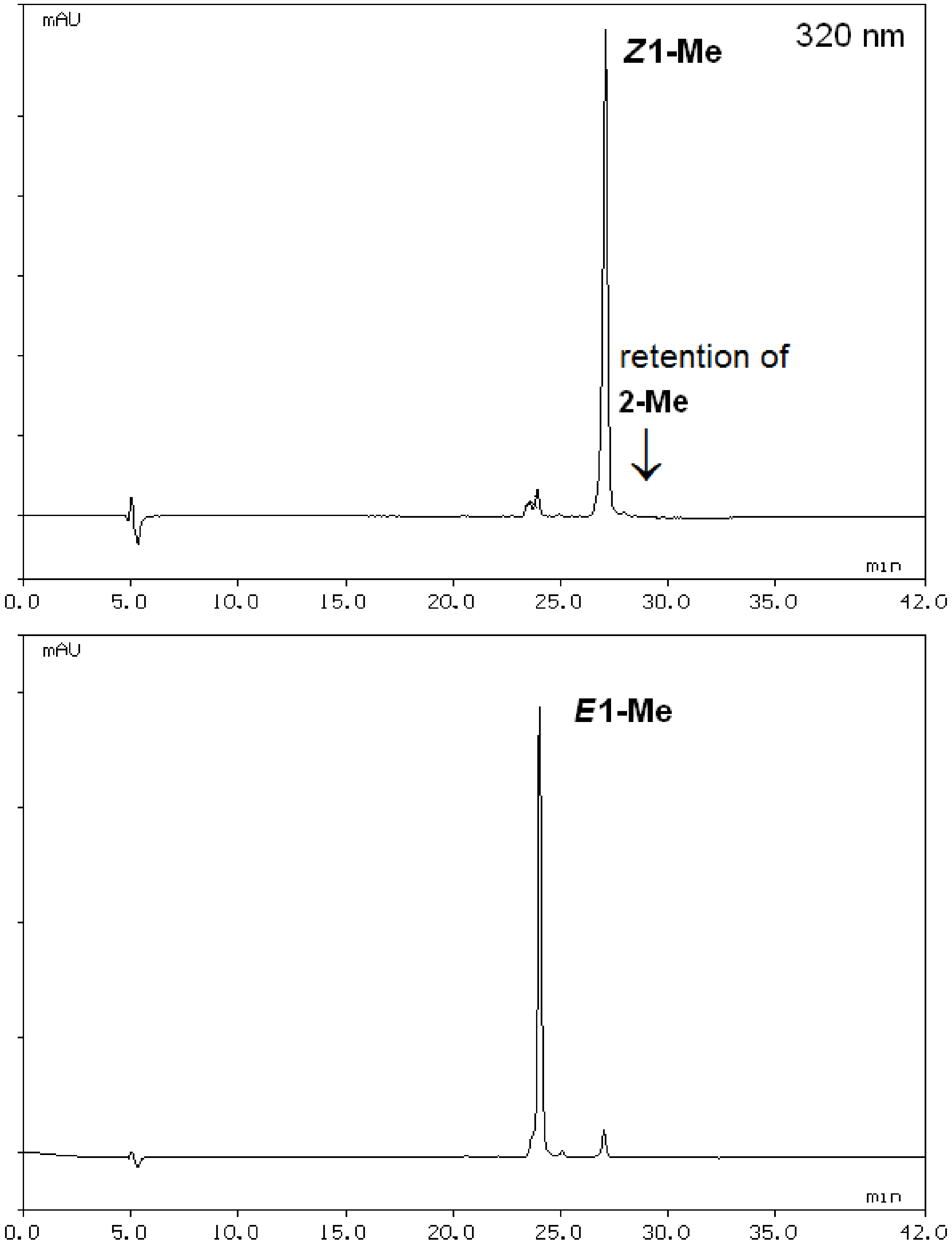


**Figure S6b.** E/Z-isomerization in the dark of a solution of ***E*1-Me** ($2.0 \times 10^{-4}$ M) in $CHCl_3$. HPLC-analysis (Sosy II) before (bottom) and after treatment (top) with 1% AcOH for 5 hours at 23°C.

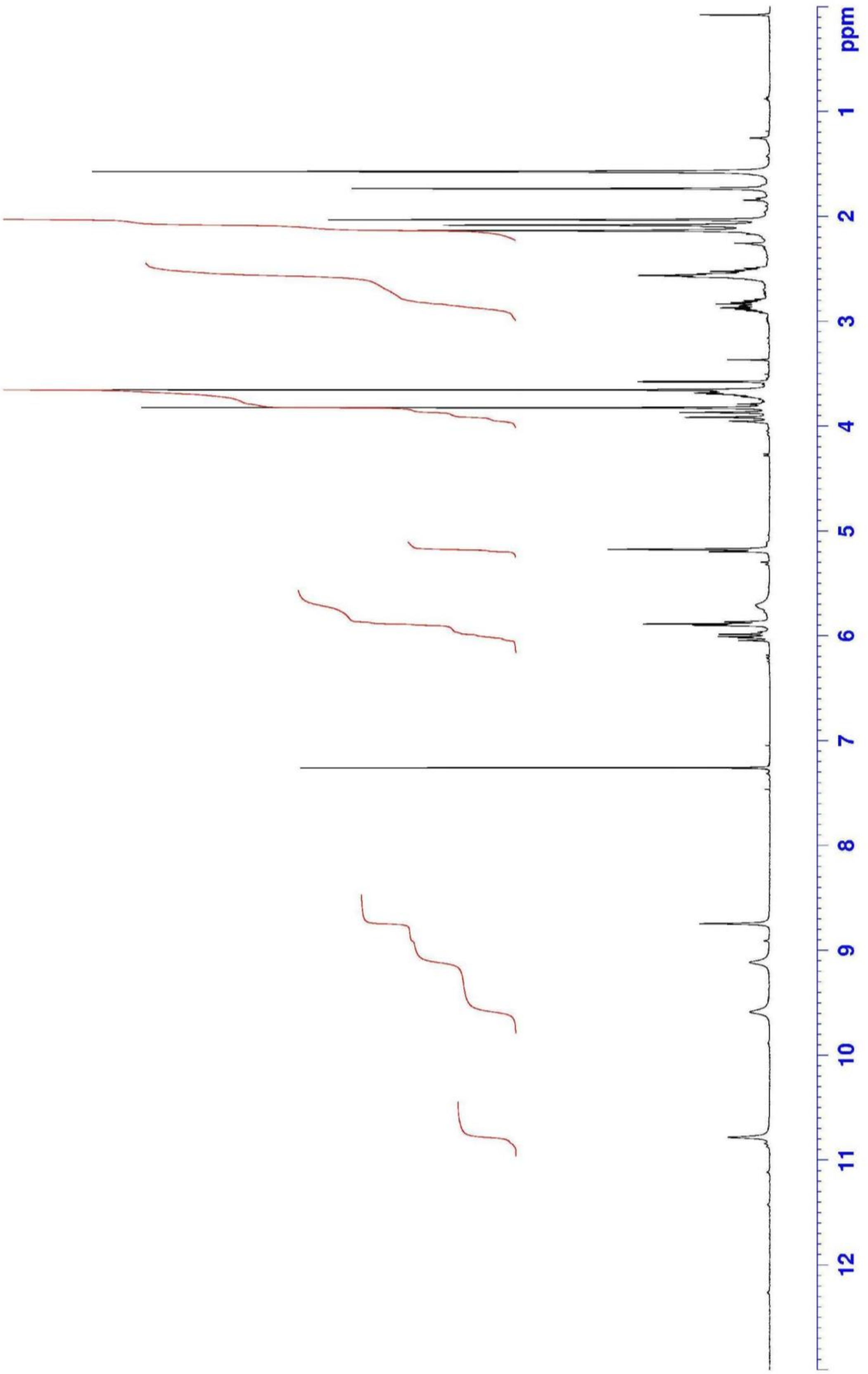


**Figure S6c.** 500 MHz $^{1}$H NMR spectrum of ***E*1-Me** in $CDCl_3$ ($4.6 \times 10^{-3}$ M, 25 °C)

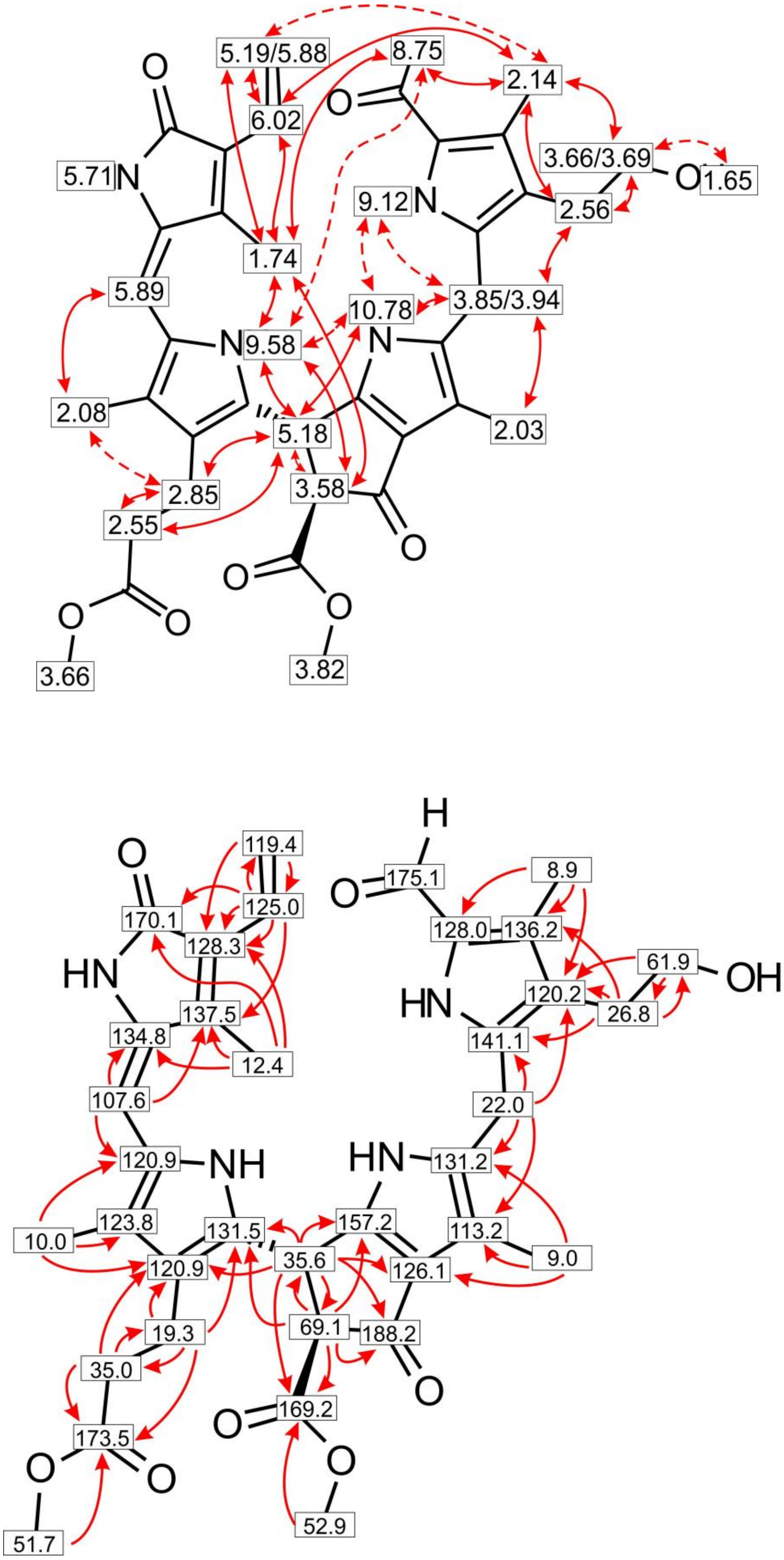


**Figure S6d.** Structural analysis of ***E*1-Me** by NMR (500 MHz, $CDCl_3$, 25°C). Top: $^{1}H,^{1}H$ homonuclear correlations from a ROESY spectrum. Bottom: $^{1}H,^{13}C$ heteronuclear correlations from HSQC- and HMBC-spectra.

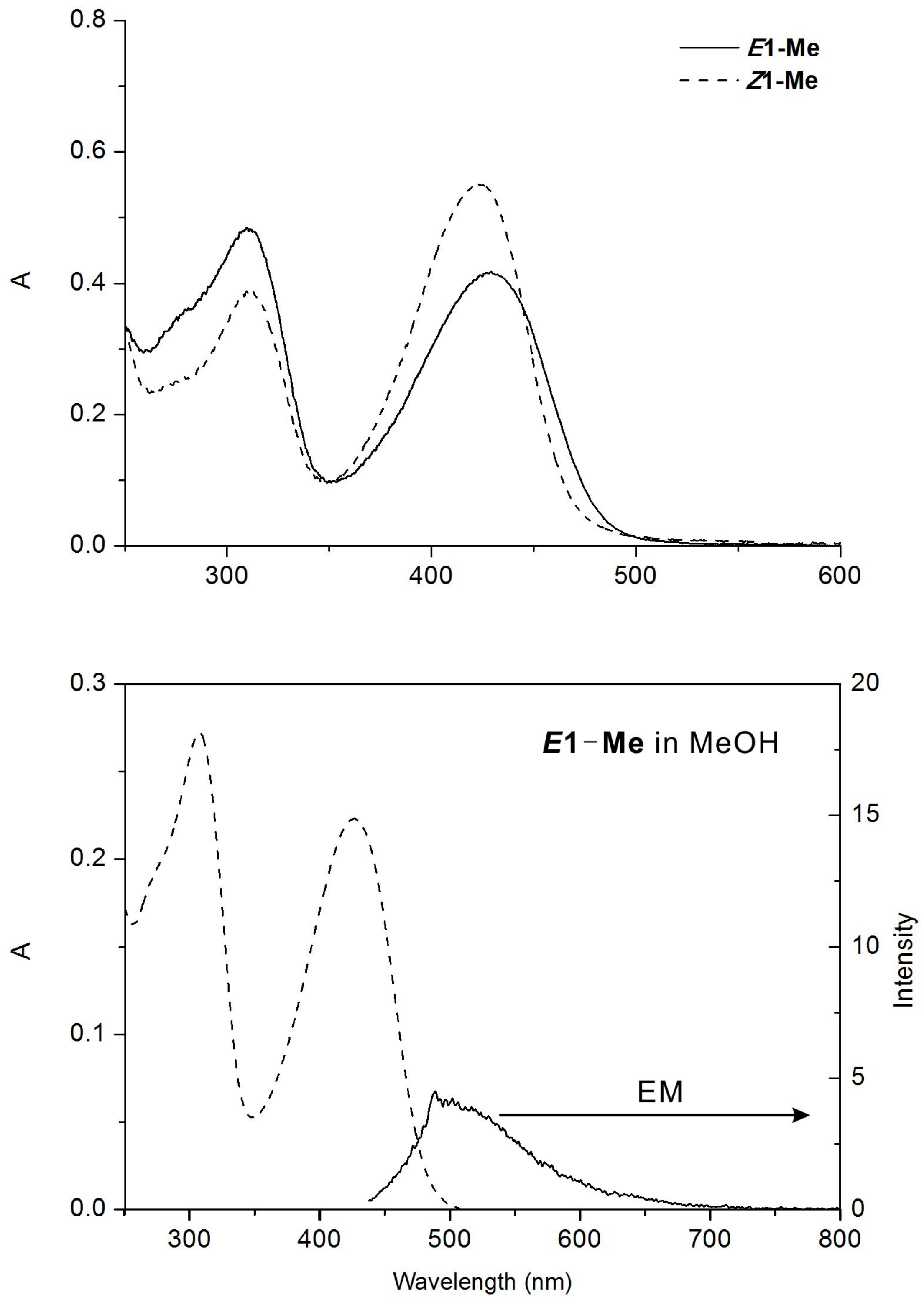


**Figure S6e.** Top: UV/Vis spectra of ***E*1-Me** ($2.3 \times 10^{-5}$ M) and ***Z*1-Me** ($1.4 \times 10^{-5}$ M) in MeOH. Bottom: UV/Vis- (dashed line) and fluorescence emission (EM) spectra (solid line) of ***E*1-Me** ($1.2 \times 10^{-5}$ M) in MeOH.

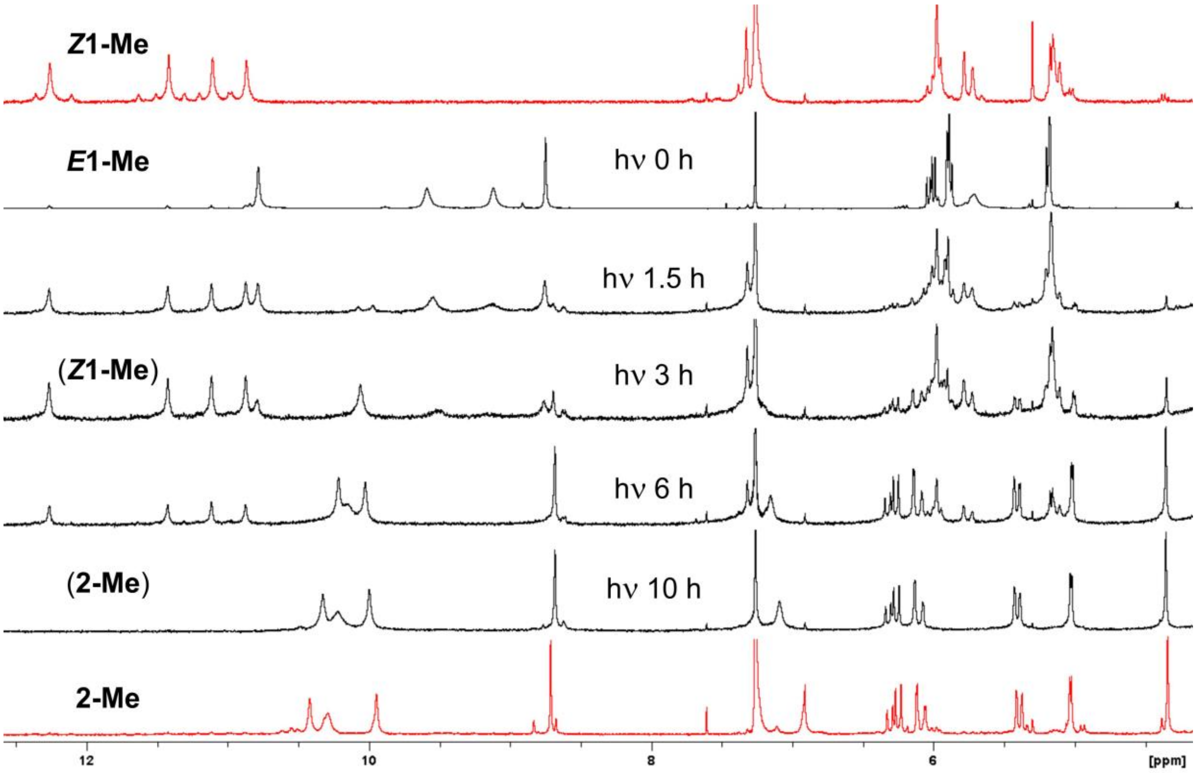


**Figure S7.** Irradiation of ***E*1-Me** ($3.0 \times 10^{-3}$ M) in $CDCl_3$ by the fluorescent lamp at 0 °C furnishes **2-Me** via ***Z*1-Me**. Reaction progress analyzed by 300 MHz $^1$H-NMR spectra, recorded at 25 °C (black lines). For comparison, the spectra of ***Z*1-Me** ($3.0 \times 10^{-3}$ M) and of **2-Me** ($1.5 \times 10^{-3}$ M) in $CDCl_3$ are shown (red lines at top and at bottom, respectively).

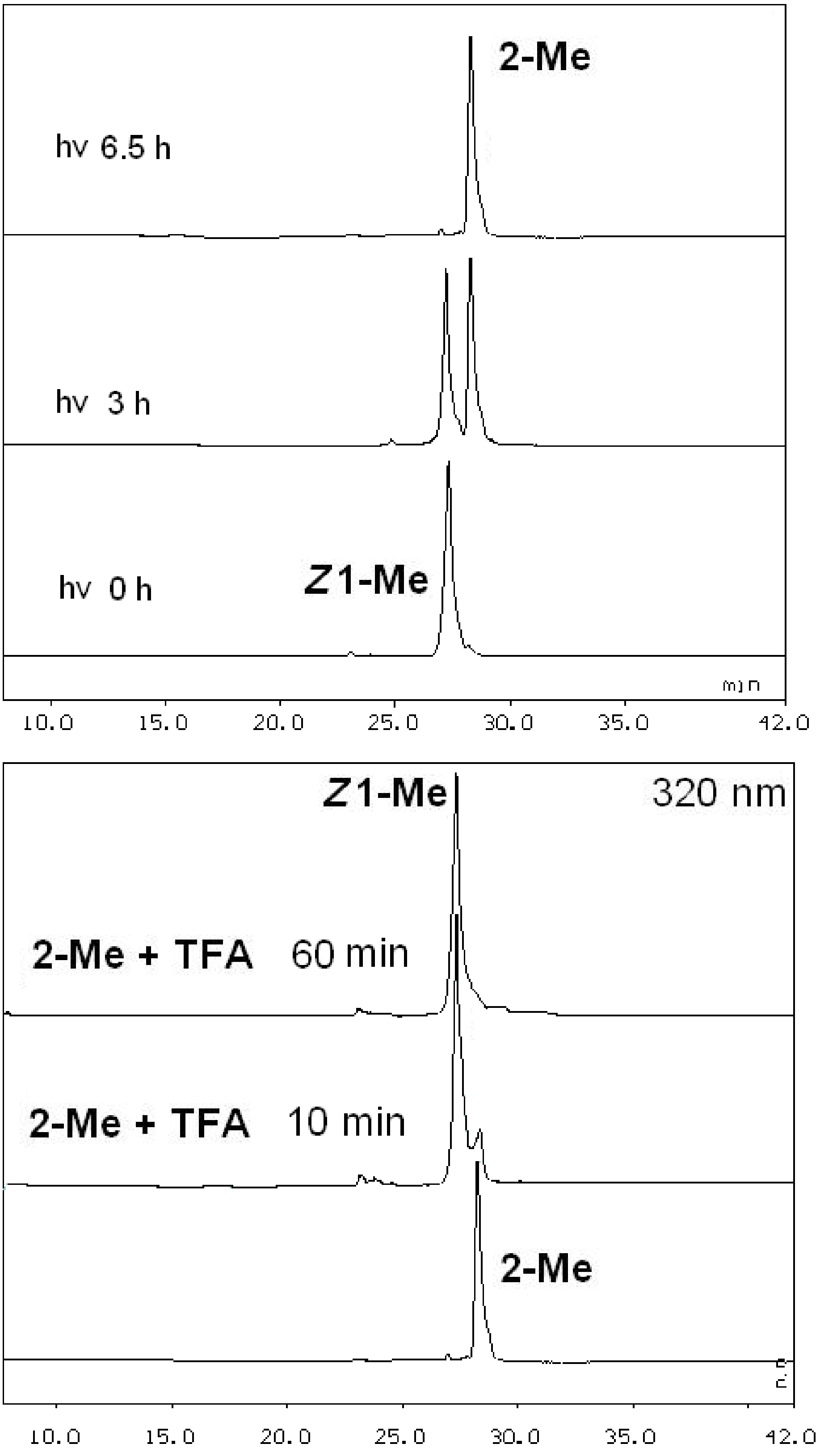


**Figure S8a.** Top: Analysis of photodimerization of ***Z*1-Me** ($1.4 \times 10^{-4}$ M) in Ar-purged $CHCl_3$ by irradiation by the fluorescent light at 0 °C, using HPLC (Sosy II). Bottom: Analysis of the acid induced cleavage of **2-Me** in $CHCl_3$ (2.8 mL, $7.0 \times 10^{-5}$ M, treated with 5 µL of trifluoroacetic acid at 23 °C in the dark), using HPLC (Sosy II), and indicated to furnish ***Z*1-Me** nearly quantitatively after 60 min.

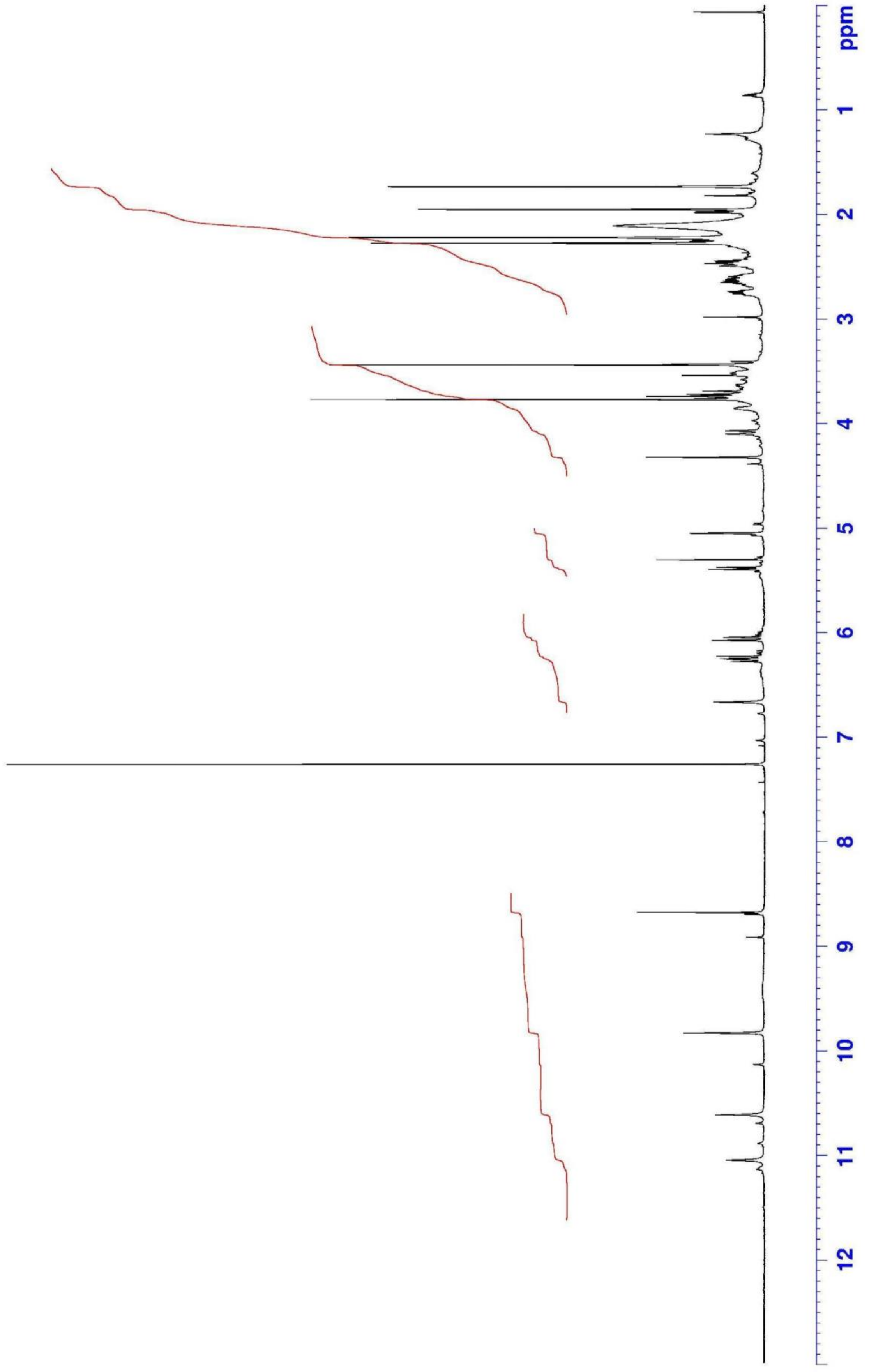


**Figure S8b.** $^{1}$H NMR spectrum of **2-Me** in $CDCl_3$ ($4.6 \times 10^{-3}$ M, 600 MHz, 2 °C)

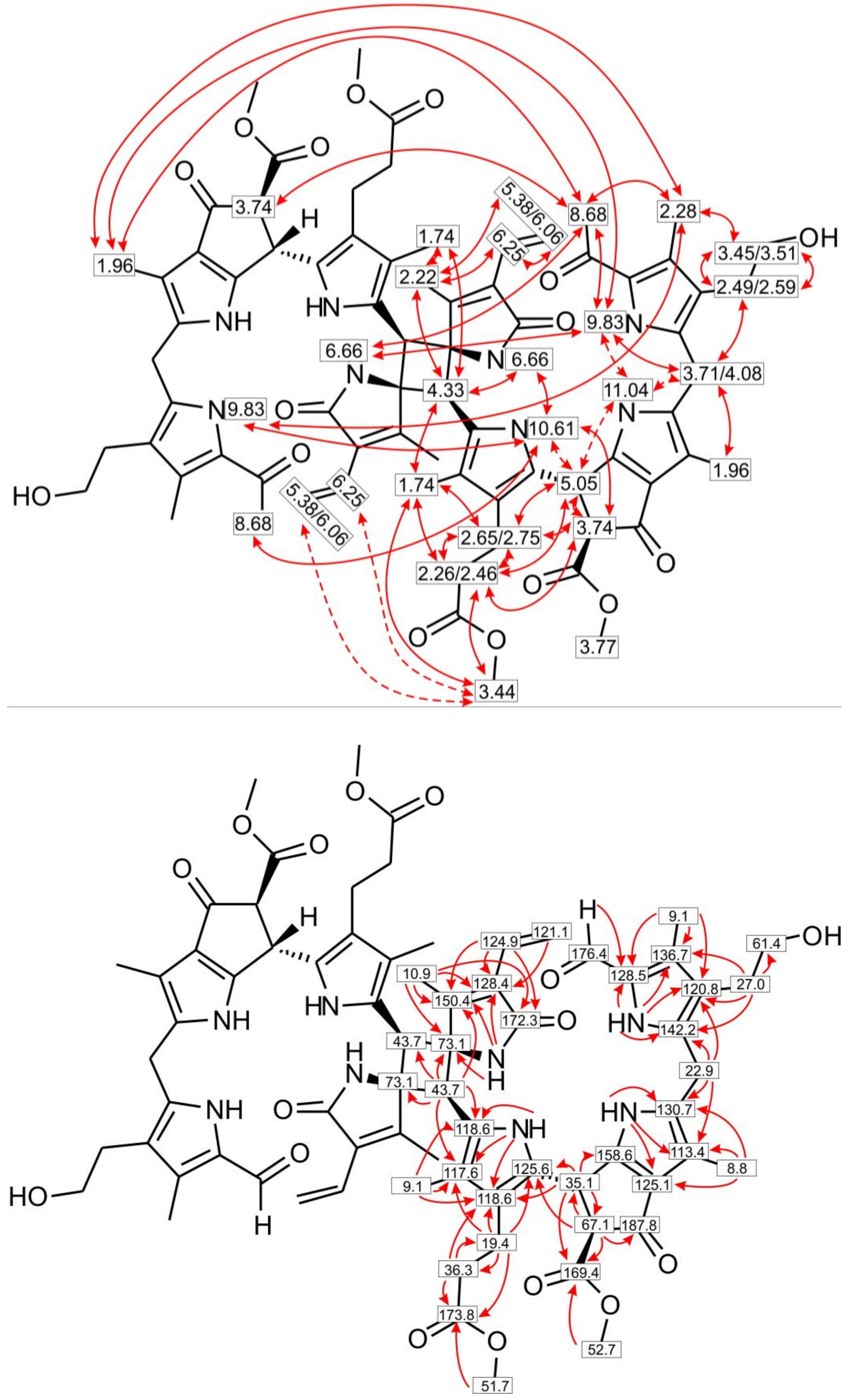


**Figure S8c.** Structure analysis of covalent homodimer **2-Me** by $^{1}$H,$^{1}$H-homonuclear and $^{1}$H,$^{13}$C-heteronuclear NMR spectra in $CDCl_3$ (at 600 MHz, 2°C). Top: Set of homonuclear correlations from a $^{1}$H,$^{1}$H-ROESY spectrum. Bottom: set of heteronuclear correlations from $^{1}$H,$^{13}$C-HSQC and $^{1}$H,$^{13}$C-HMBC spectra.

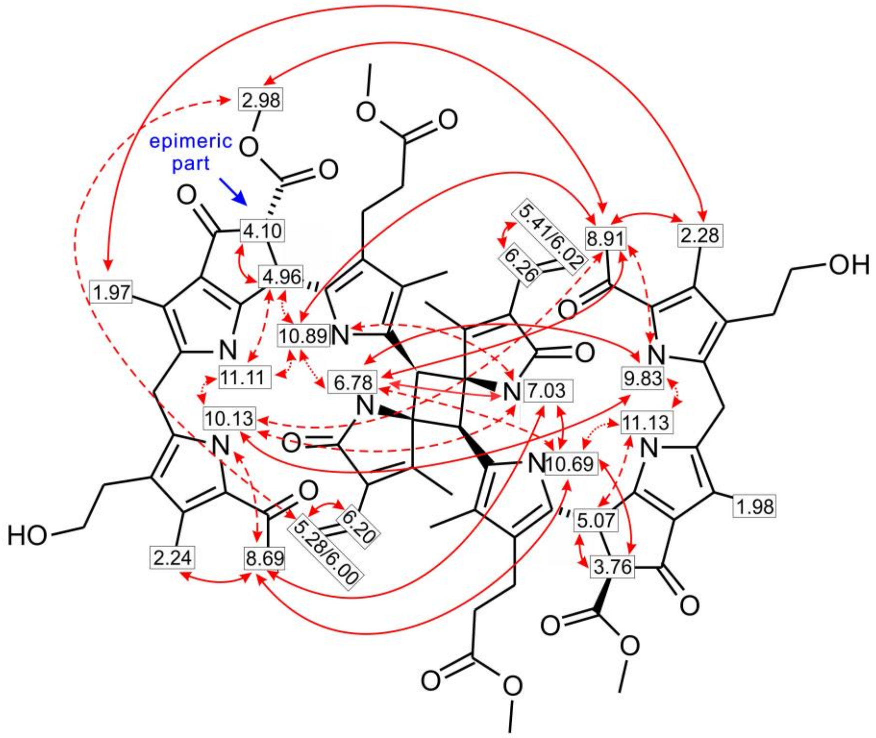


**Figure S8d.** Homonuclear correlations of covalent heterodimer ***epi*-2-Me** from a 600 MHz $^{1}H,^{1}H$ ROESY spectrum ($CDCl_3$, 2°C).

**Table S5** $^{1}H$-NMR signal assignments of covalent dimers **2-Me** and ***epi*-2-Me** in $CDCl_3$.

| | **2-Me** [a] | normal module in ***epi*-2-Me** [a] | epimeric module in ***epi*-2-Me** [a] |
|---|---|---|---|
| $H_3C$-$2^1$ | 2.28 (s) | 2.28 (s) | 2.24 (s) |
| $H_2C$-$3^1$ | 2.49 / 2.59 (m) | | |
| $H_2C$-$3^2$ | 3.45 / 3.51 (m) | | |
| $H_2C$-5 | 3.71 / 4.08 ( AB, 19.1 Hz) | | |
| $H_3C$-$7^1$ | 1.96 (s) | 1.98 (s) | 1.97 (s) |
| HC-$8^2$ | 3.74 (d, 3.7 Hz) | 3.76 (d, 3.8 Hz) | 4.10 (d, 7.5Hz) |
| $H_3C$-$8^5$ | 3.77 (s) | | 2.98 |
| HC-10 | 5.05 (d, 3.7 Hz) | 5.07 (d, 3.8 Hz) | 4.96 (d, 7.5 Hz) |
| $H_2C$-$12^1$ | 2.65 / 2.75 (m) | | |
| $H_2C$-$12^2$ | 2.26 / 2.45 (m) | | |
| $H_3C$-$12^5$ | 3.44 (s) | | |
| $H_3C$-$13^1$ | 1.74 (s) | | |
| HC-15 | 4.33 (s) | | |
| $H_2C$-$17^1$ | 2.22 (s) | | |
| HC-$18^1$ | 6.25 (dd, 11.6 / 17.6 Hz) | 6.26 | 6.20 |
| $H_3C$-$18^2$ | 5.38 (dd, 1.3 / 11.6 Hz)<br>6.06 (dd, 1.3 / 17.6 Hz) | 5.41<br>6.02 | 5.28<br>6.00 |
| HC-20 | 8.68 (s) | 8.91 (s) | 8.69 (s) |
| HN21 | 9.83 (s) | 9.83 (s) | 10.13 (s) |
| HN22 | 11.04 (s) | 11.13 (s) | 11.11 (s) |
| HN23 | 10.61 (s) | 10.69 (s) | 10.89 (s) |
| HN24 | 6.66 (s) | 7.03 (s) | 6.78 (s) |

[a] chemical shift (signal type, coupling constant)

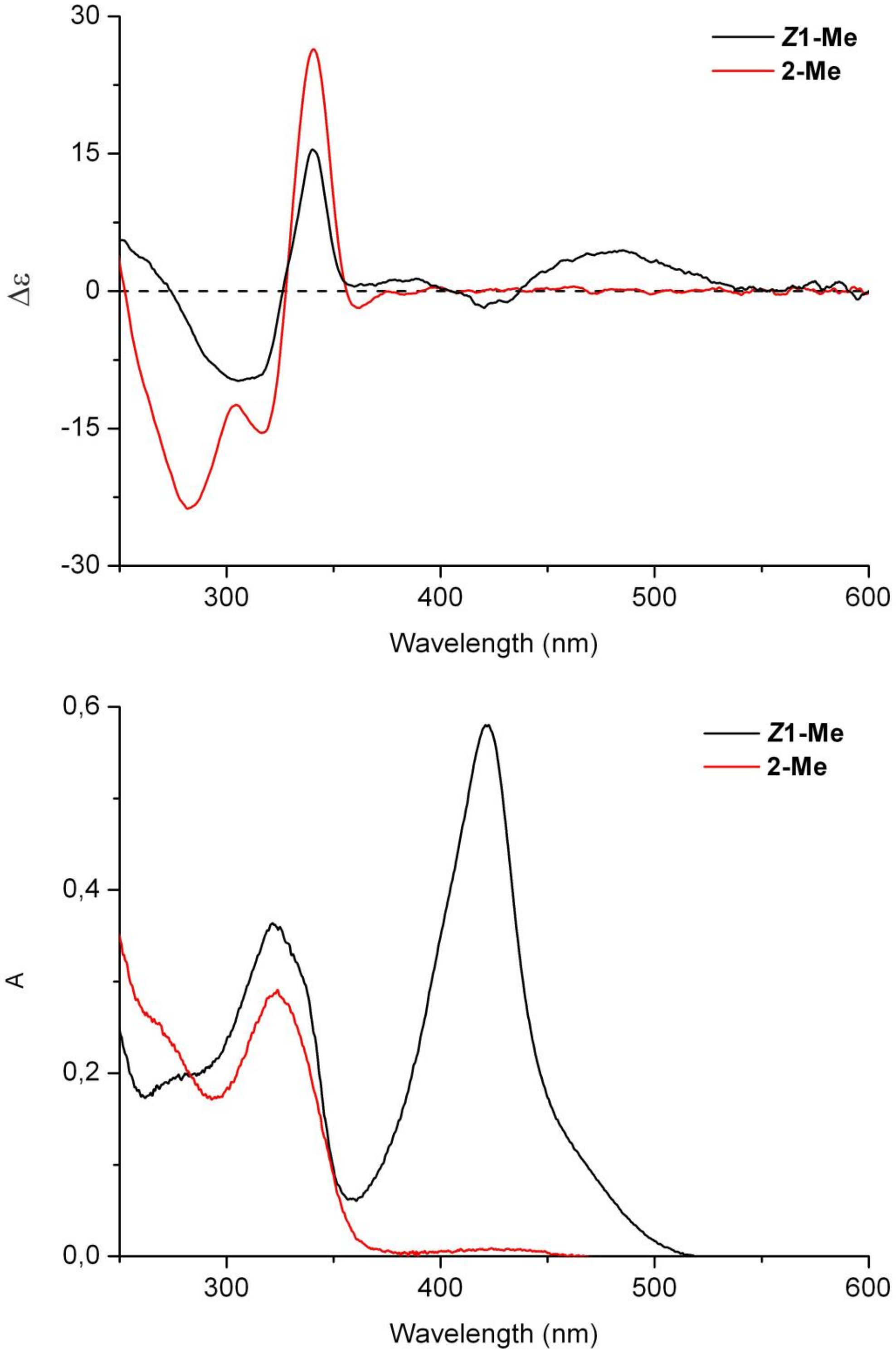


**Figure S8e.** CD-spectra (top) of ***Z*1-Me** ($2.5 \times 10^{-5}$ M) and **2-Me** ($1.25 \times 10^{-5}$ M) and UV/Vis-spectra (bottom) of ***Z*1-Me** ($1.4 \times 10^{-5}$ M) and **2-Me** ($7.0 \times 10^{-6}$ M**)** in $CHCl_3$**.**

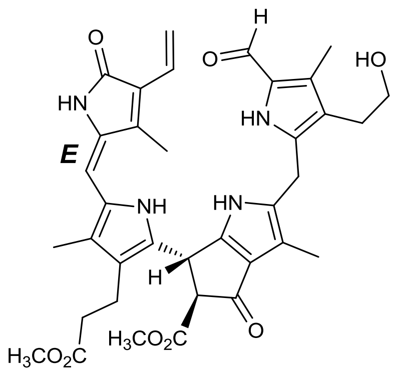


***E*1-Me**

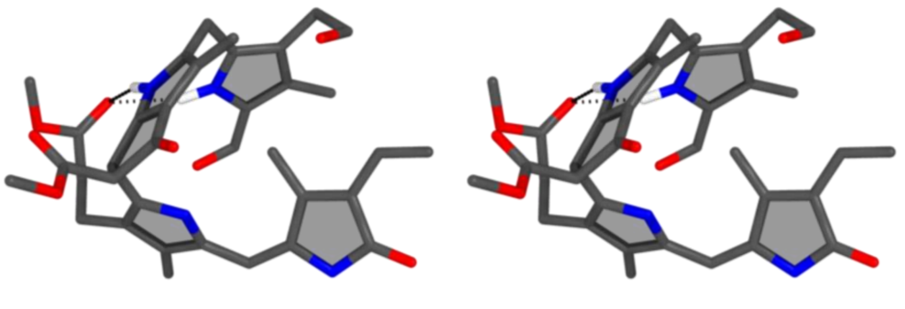

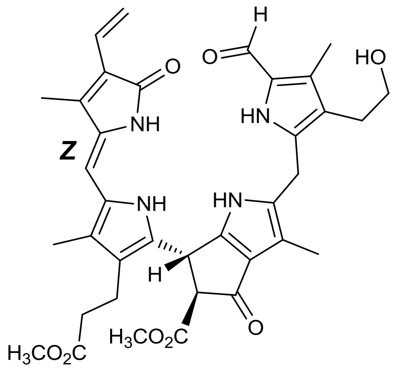


***Z*1-Me**

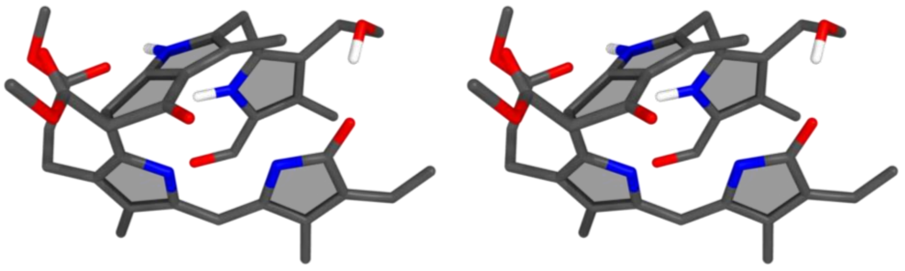

**Figure S9a.** Stereo-projection of four calculated models of the ***E*1-Me** (top) and ***Z*1-Me** (bottom) (dashed line: H-bonds; color code: C gray, O red, N blue, H white).

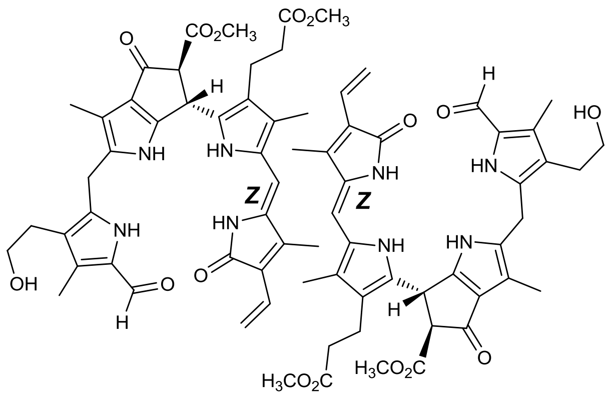

**(*Z*1-Me)$_2$**

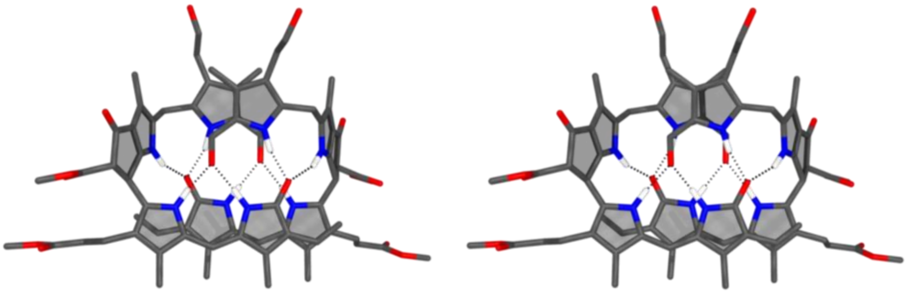

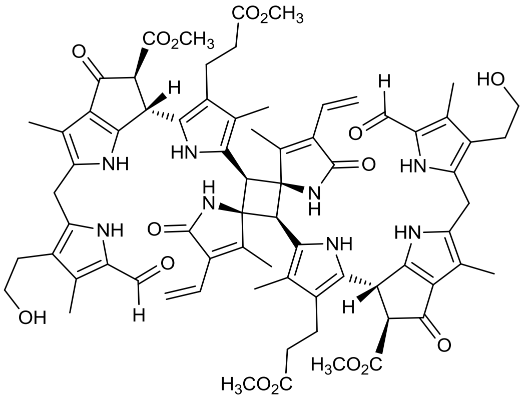

**2-Me**

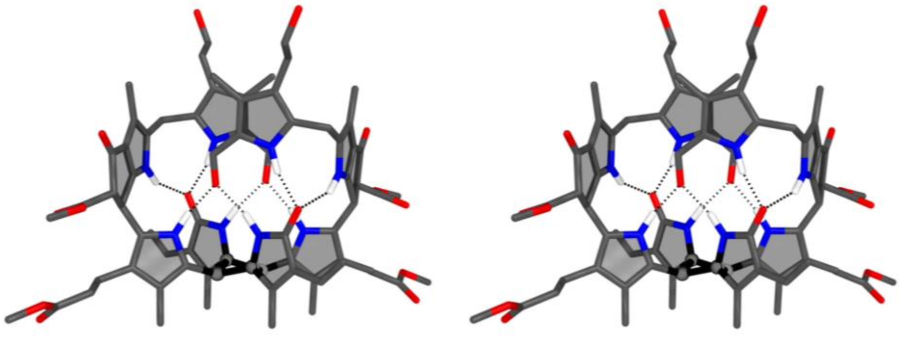

**Figure S9b.** Stereo-projection of four calculated models of the **(*Z*1-Me)$_2$** (top) and **2-Me** (bottom) (dashed line: H-bonds; color code: C gray, O red, N blue, H white).

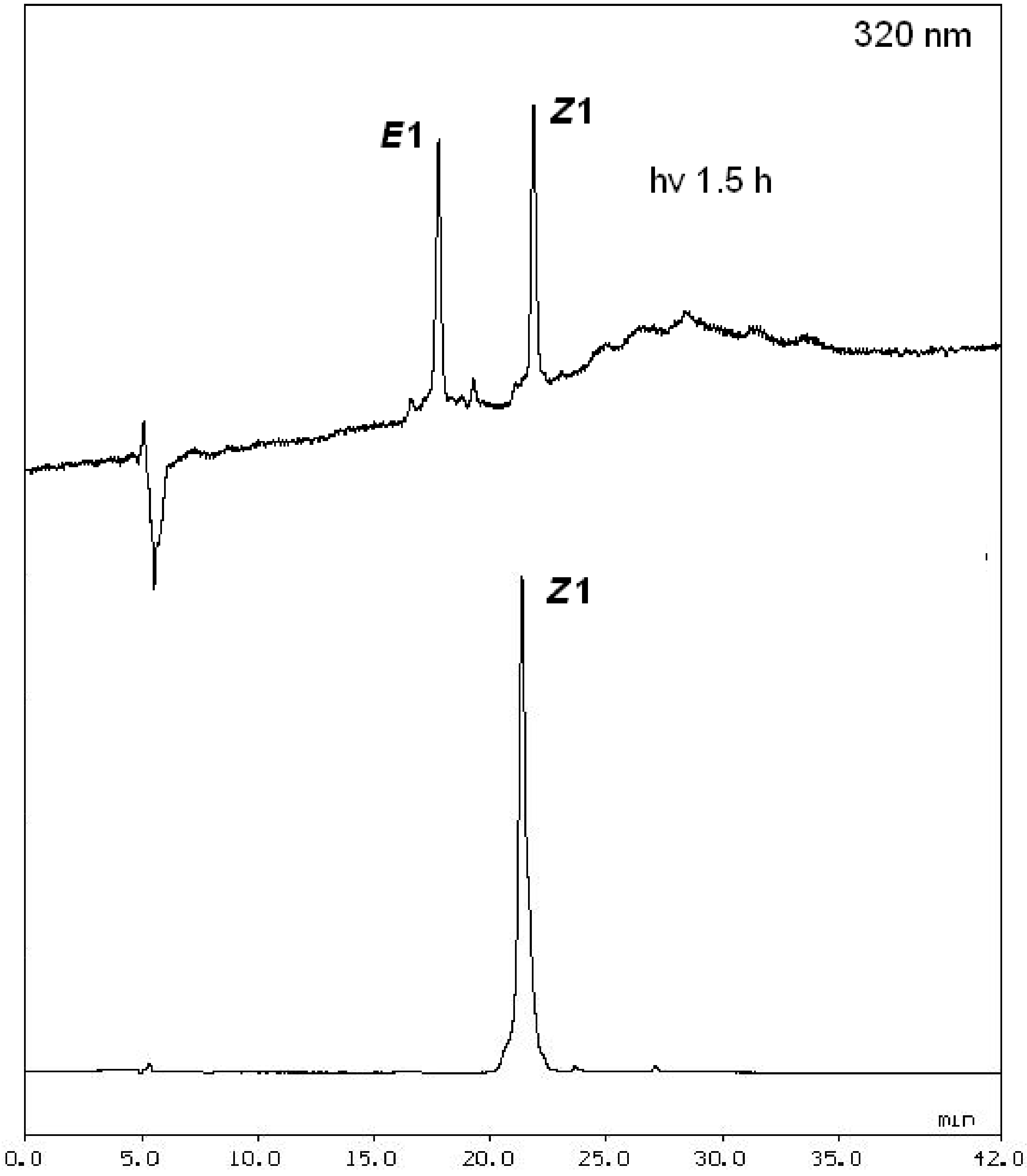


**Figure S10a.** Z/E-photoisomerization of ***Z*1** (2.5 × $10^{-6}$ M) in 50 mM aqueous potassium phosphate buffer (pH 7) upon illumination by the fluorescent lamp (HPLC-analysis, Sosy II).

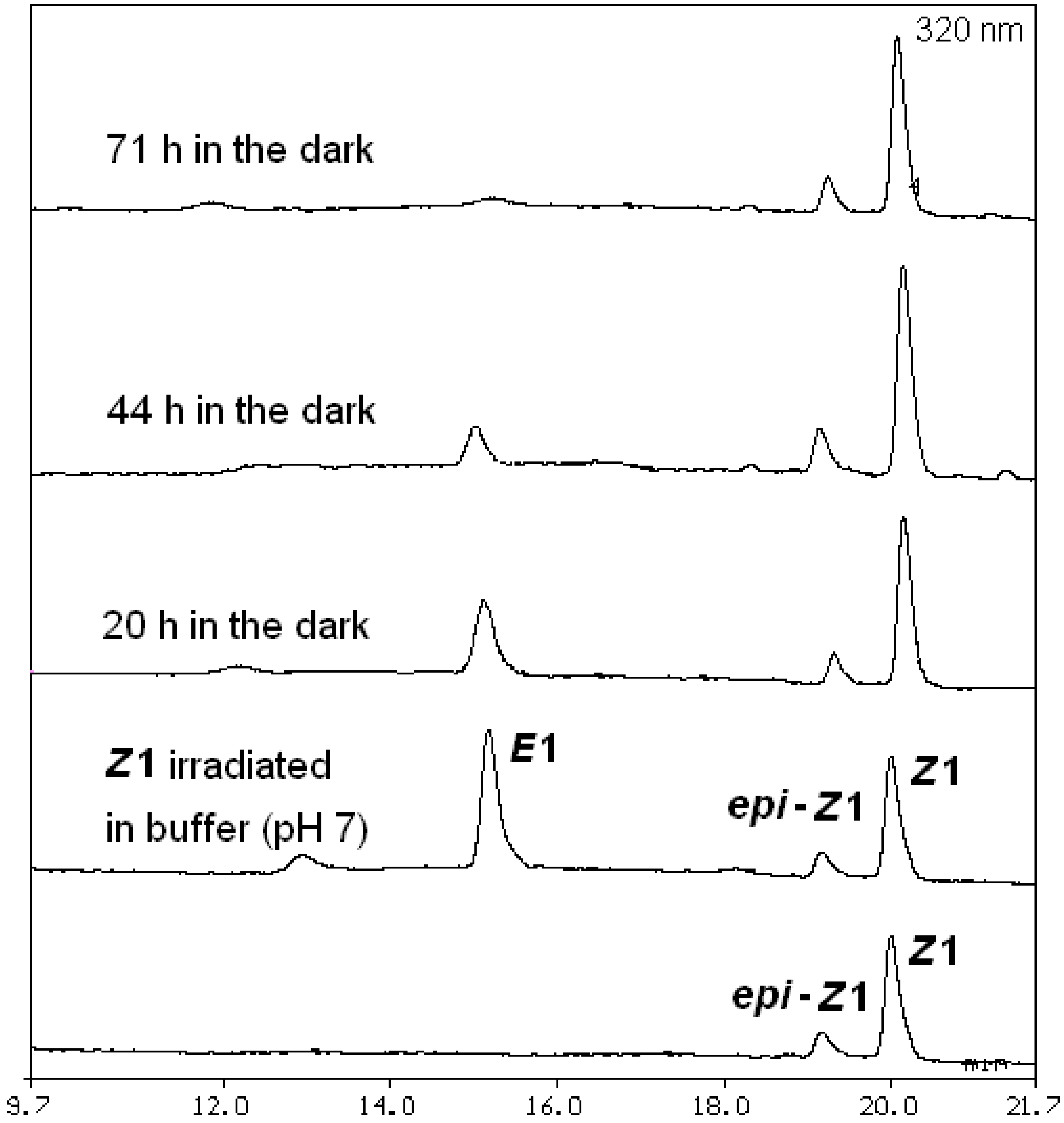


**Figure S10b.** Z/E-Photoisomerization of ***Z*1** (7.0 × $10^{-6}$ M in 50 mM aqueous potassium phosphate buffer, pH 7) after irradiation for 160 minutes by the fluorescent lamp, followed by thermal E/Z-isomerization in the dark; HPLC-analysis (Sosy III) after the irradiation and after subsequent storage of the solution for 20, 44 and 71 h at 23 °C (during overnight stirring of the starting mixture with pure ***Z*1**, required in order to dissolve ***Z*1** to the desired concentration, **Z1** epimerized to ***epi*-*Z*1**, in part, see lowest trace).

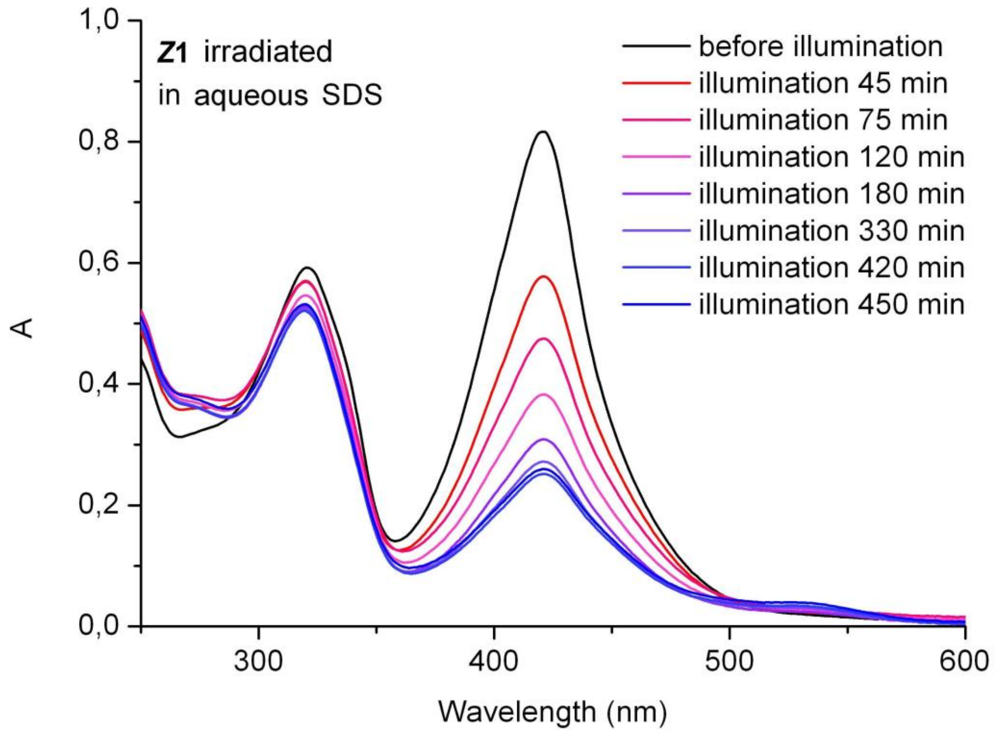


**Figure S11a.** UV/Vis spectroscopic analysis of photoreaction of ***Z*1** ($2.6 \times 10^{-4}$ M) in aqueous 16.4 mM SDS, when irradiated by the fluorescent lamp at 23 °C.

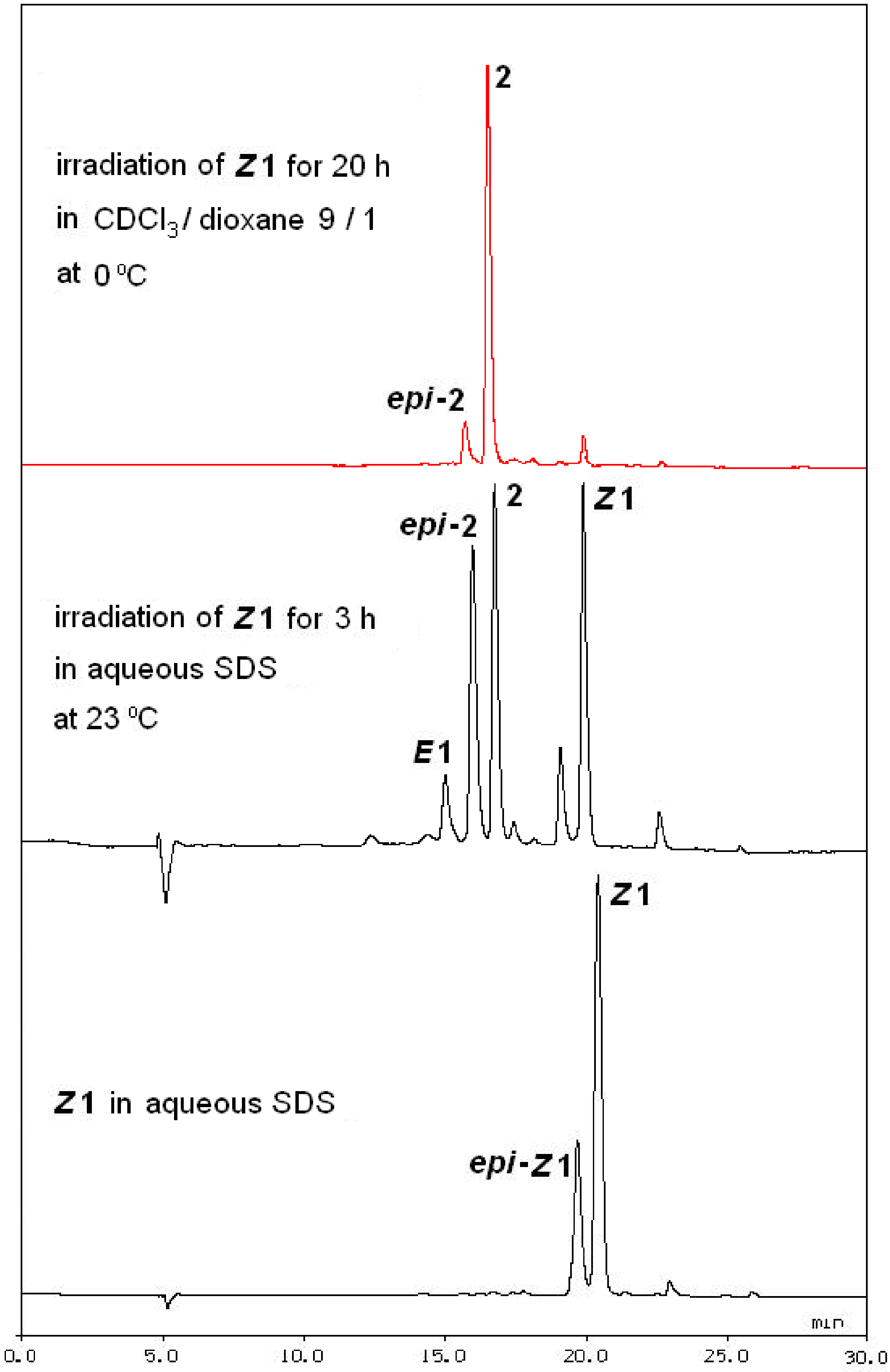


**Figure S11b.** Photodimerization of ***Z*1** ($1.6 \times 10^{-3}$ M) in Ar-purged $CDCl_3$ / dioxane (9 / 1, v / v)); HPLC-analysis (Sosy III) after irradiation for 20 hours by the fluorescent lamp at 0 °C. Photoreaction of ***Z*1** ($2.6 \times 10^{-4}$ M, in aqueous SDS); HPLC-analysis (Sosy III) after irradiation for 3 hours by the fluorescent lamp at 23 °C. (during overnight stirring of the starting mixture with pure ***Z*1**, required in order to dissolve ***Z*1** to the desired concentration in aqueous SDS, ***Z*1** epimerized to ***epi*-*Z*1**, in part, see lowest trace).

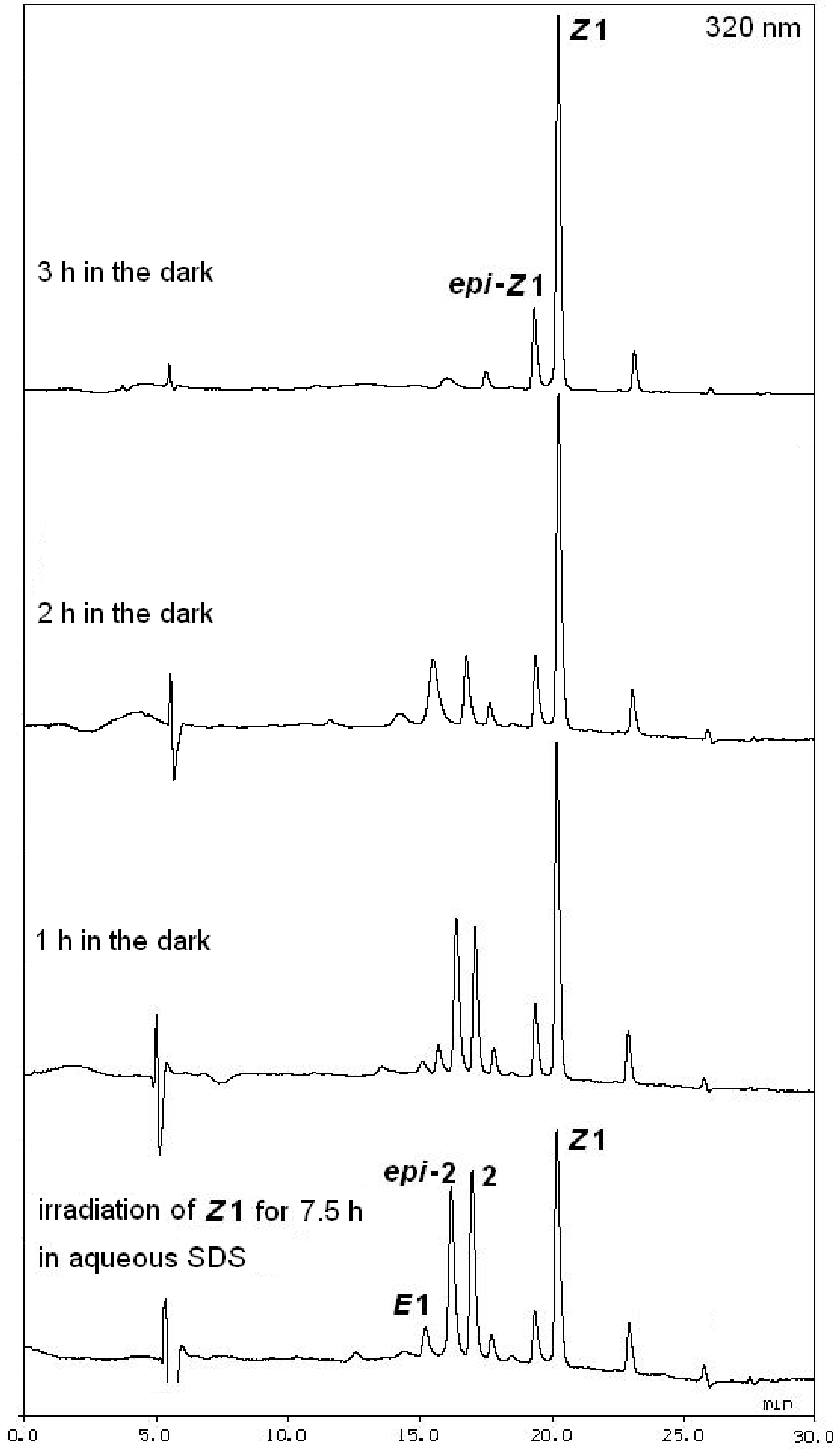


**Figure S11c.** Photoreaction of ***Z*1** ($2.6 \times 10^{-4}$ M in aqueous SDS) after irradiation for 7.5 hours by the fluorescent lamp at 23 °C, followed by thermal reverse reaction in the dark; HPLC-analysis (Sosy III) after the irradiation and after subsequent storage of the solution for 1, 2 and 3 h in the dark at 23 °C.

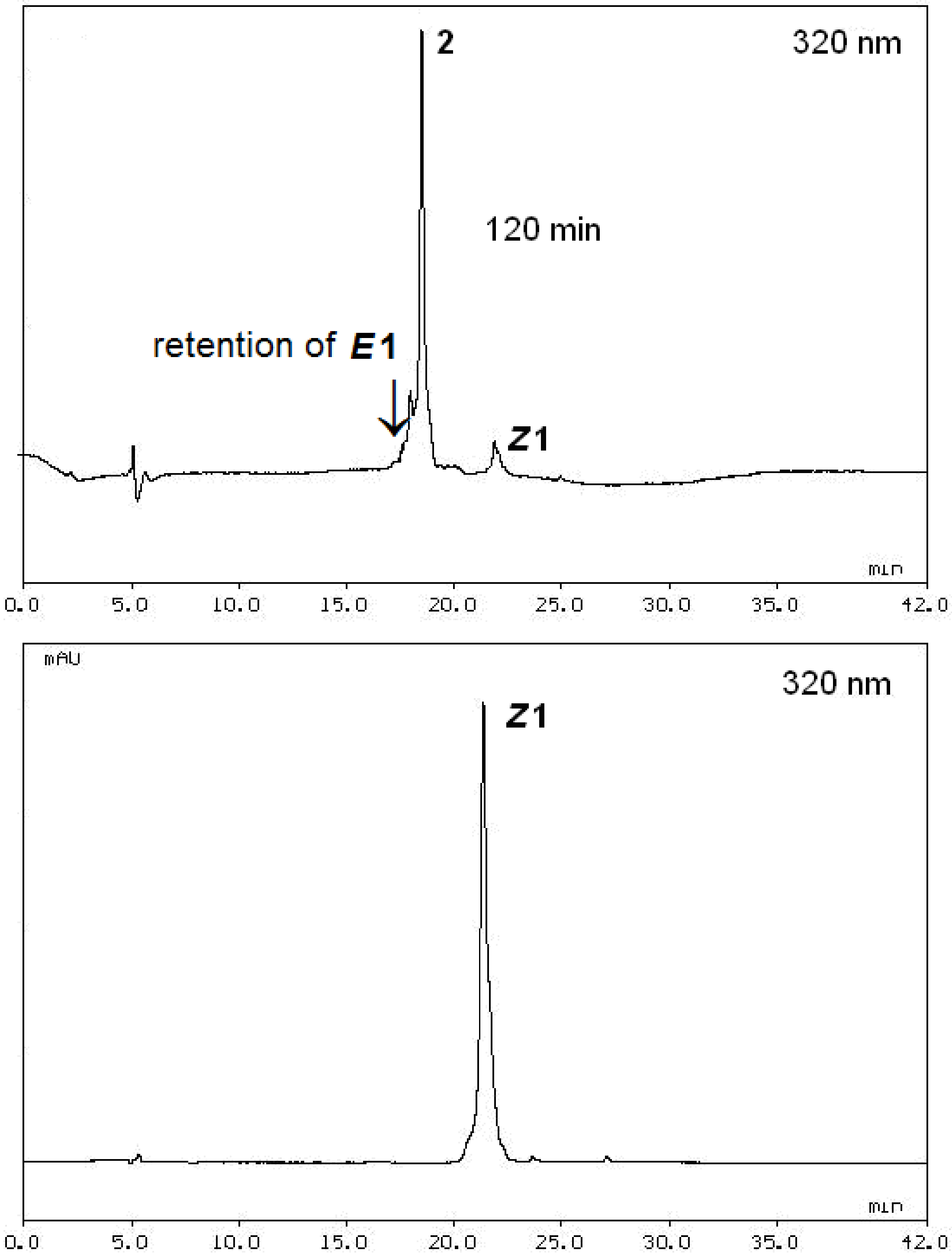


**Figure S12a.** Photodimerization of ***Z*1** ($3.0 \times 10^{-4}$ M) to **2** in Ar-purged $CHCl_3$/dioxane (99/1, v/v). HPLC-analysis (Sosy II) before (bottom) and after irradiation (top) by the fluorescent lamp at 0 °C.

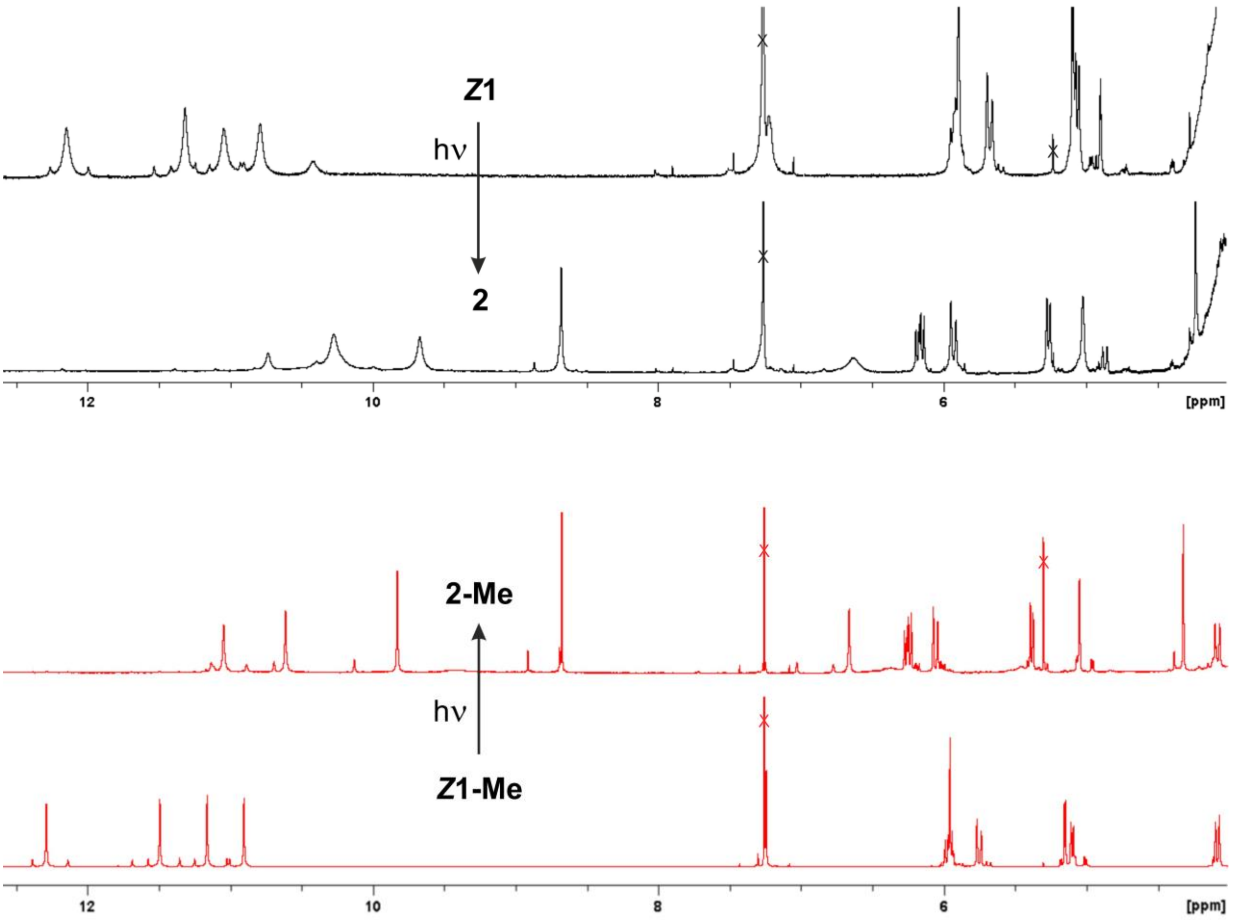


**Figure S12b.** NMR-Analysis of the photodimerization of ***Z*1** to **2** (top) and of ***Z*1-Me** to **2-Me** (bottom). Top: 500 MHz $^{1}$H NMR spectra of ***Z*1** ($1.6 \times 10^{-3}$ M, $CDCl_3$ / dioxane, 9/1, v/v, at 25°C), and of **2** ($8 \times 10^{-4}$ M, $CDCl_3$ / dioxane, 9/1, v/v, at 0°C) obtained from irradiation of ***Z*1** in $CDCl_3$ / dioxane. Bottom: 600 MHz $^{1}$H NMR spectra of ***Z*1-Me** ($1.0 \times 10^{-2}$ M, $CDCl_3$, at 2°C) and **2-Me** ($4.6 \times 10^{-3}$ M, $CDCl_3$, at 2°C) obtained from irradiation of ***Z*1-Me** in $CDCl_3$. X's mark solvent signals.

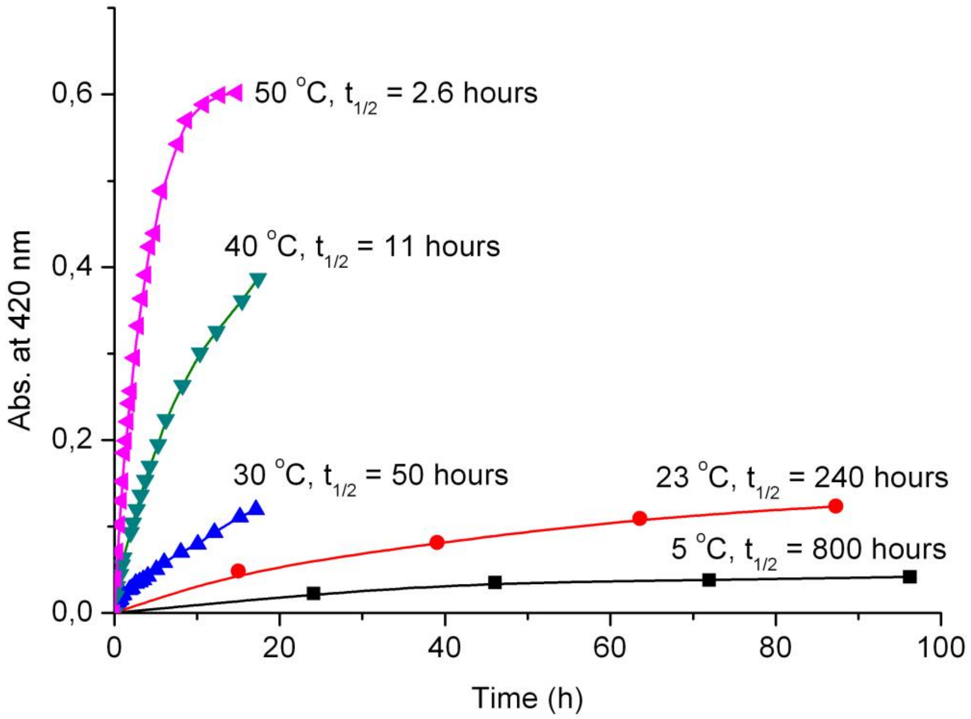


**Figure S13.** Decomposition of **2-Me** ($8.3 \times 10^{-5}$ M) in Ar-purged acid free $CHCl_3$ in the dark and at the indicated temperatures and formation of ***Z*1-Me**, monitored by the increase of the absorption near 420 nm.

## References

[1] C. Li, B. Kräutler,. *J Porph & Phthalocyanines* **2016**, *20*, 388-396.
[2] S. Moser, M. Ulrich, T. Müller, B. Kräutler, *Photochem. Photobiol. Sci.* **2008**, *7*,1577-1581.
[3] V6.6 T. A development of University of Karlsruhe and Forschungszentrum Karlsruhe GmbH, 1989-2007 (since 2007: TURBOMOLE GmbH), **2014**.
[4] A. D. Becke, *Phys. Rev. A.* **1988**, *38*, 3098-3100.
[5] K. Eichkorn, O. Treutler, H. Öhm, M. Häser, R. Ahlrichs, *Chem. Phys. Lett.* **1995**, *240*, 283-290.
[6] F. Weigend, R. Ahlrichs, *Phys. Chem. Chem. Phys.* **2005**, *7*, 3297-3305.
[7] S. Grimme, J. Antony, S. Ehrlich, H. Krieg, *J. Chem. Phys.* **2010**, *132***,** 154104.
[8] Schrödinger Release 2015-2: Maestro, version 10.2, Schrödinger, LLC, New York, **2015**.
[9] SHELXTL v2014/4, Bruker AXS Inc., Madison, Wisconsin, USA.
[10] G.M.Sheldrick, *Acta Cryst* **2015**, *c71*, 3-8.
[11] The PyMOL Molecular Graphics System, Version 1.8
